# Phytochemicals of *Alpinia zerumbet*: A Review

**DOI:** 10.3390/molecules29122845

**Published:** 2024-06-14

**Authors:** Yuto Nishidono, Ken Tanaka

**Affiliations:** 1 College of Pharmaceutical Sciences, Ritsumeikan University, Kusatsu 525-8577, Shiga, Japan; ktanaka@fc.ritsumei.ac.jp; 2 Research Organization of Science and Technology, Ritsumeikan University, Kusatsu 525-8577, Shiga, Japan

**Keywords:** *Alpinia zerumbet*, *Alpinia speciosa*, shell ginger, multiple medicinal parts, kavalactones

## Abstract

*Alpinia zerumbet* (Pers.) B.L.Burtt & R.M.Sm is a perennial plant of the *Zingiberaceae* family widely distributed in the subtropical and tropical areas of South America, Oceania, and Asia. Multiple plant parts of *A. zerumbet* have been traditionally used as medicinal sources, each with different clinical uses. These variations may arise from differences among the chemical components and/or accumulations of the active compounds in each part. Therefore, this review summarizes previous studies on the phytochemicals in *A. zerumbet* and reveals the similarities and differences among the chemical constituents of its multiple medicinal parts, including the leaves, rhizomes, fruits, seeds, and flowers. The results contribute to the scientific validation of the traditional understanding that *A. zerumbet* possesses different medicinal properties in each plant part. In addition, this review provides directions for further studies on the phytochemicals of this plant.

## 1. Introduction

*Alpinia zerumbet* (Pers.) B.L.Burtt & R.M.Sm, also known as shell ginger, is a perennial species belonging to the *Zingiberaceae* family. The previously accepted scientific name of shell ginger, *A. speciosa* (J.C.Wendl.) K.Schum., is now recognized as a representative synonym along with *A. nutans* K.Schum. and *Catimbium speciosum* (J.C.Wendl.) Holttum [1]. Shell ginger originated from the East Indies [2] and is native in areas ranging from South Japan to Taiwan and from South China to the Northern Peninsula [1]. The plant has also been introduced to Brazil, where it has since naturalized [3]. Therefore, *A. zerumbet* is currently found in the subtropical and tropical areas of South America, Oceania and Asia [4] and is variously known as *Vindicá* (Amazon) [5], *Colônia* (Brazil) [6], *Yàn Shān jiāng* (China) [7], yellow ginger (Fiji) [8], *Chatiun* in Hindi (India) [9], *Punnag-champa* in Bengal (India) [10], *Gettō* (Japan) [11], *Sannin* (Okinawa, Japan) [11], *Atoumo* (Martinique, French West Indies) [12], *Yuetao* (Taiwan) [1], and *Riềng đẹp* (Vietnam) [13].

In the endemic regions of *A. zerumbet*, various parts of the plant have been used for various purposes. The leaves, rhizomes, fruits, seeds, and flowers of *A. zerumbet* have been traditionally used for medicinal purposes. In Brazil, a decoction of the leaves is believed to exhibit an anti-hemorrhoidal activity [14]. Infusions, decoctions, and teas made from *A. zerumbet* leaves have been consumed as anti-hypertensives and diuretics [15,16,17]. Especially in Marajó (Brazil), the leaves have been used as remedies for colds and influenza and are added to tea and bathwater [5]. According to traditional Chinese folklore, the rhizomes of *A. zerumbet* can effectively treat trauma and peptic ulcers [18]. The rhizomes are also used as a preservative or flavoring condiment in alcoholic beverages [19], and the seeds are a popular aromatic stomachic [18,20]. The Miao people in Guizhou Province (China) have used the mature fruits (pericarps, placenta, and seeds) as cardiovascular disease treatments [7,21,22]. In India, the rhizomes have been applied to sufferers of catarrhal and rheumatism affections [23]. In Okinawa (Japan), the leaves are used in the traditional *muchi* cuisine (rice cake wrapped in *A. zerumbet* leaves), which is thought to protect against the common cold [24,25,26]. The leaves are also sold as herbal teas [24,27], and their essential oils are used in cosmetics, perfumes, insect repellents, and deodorants [4,24]. The seeds are employed as an aromatic stomachic [20,28] and were sold as *Amomum* seeds native to Japan in the first half of the twentieth century [29]. In Martinique of the French West Indies, decoctions of *A. zerumbet* flowers have been used as a bronchitis treatment [12]. Taiwanese indigenous peoples such as the Paiwan, Rukai, and Puyuma use the leaves in *abay* cuisine (leaf-covered rice with meat) [30]. The flowers are incorporated into culinary preparations. The seeds are used as medicinal ingredients [30]. In Vietnam, fever, abdominal pain, bloating, indigestion, and diarrhea are treated with a concoction obtained from boiled leaves, rhizomes, flowers, and seeds of the plant [13].

The number of studies on *A. zerumbet* has increased in recent times, revealing various bioactivities and pharmacological activities of the extracts and essential oils prepared from multiple medicinal parts of the plant (Table 1). These differences possibly arise from differences among the chemical components and/or the accumulation of active compounds in the individual medicinal parts. Therefore, by comprehensively clarifying the similarities and differences among the chemical constituents of the medicinal parts of *A. zerumbet*, we can better understand their different pharmacological activities and assess their clinical efficacies.

Five reviews on *A. zerumbet* have been written in English as of May 2024 [99,100,101,102,103]. In 2014, Devi and Rao [99] reviewed the therapeutic aspects of shell ginger. The botany, uses, phytochemistry, pharmacological properties, and clinical trials of *A. zerumbet* were reviewed by Chan et al. [100] in 2017, and the properties of this plant were briefly reviewed by Kumar and Bind [101] in 2018. Xiao et al. [102] summarized the chemical constituents in the fruits of shell ginger and elucidated their anti-atherosclerosis mechanisms. Chan et al. [103] reviewed the pharmacological properties of kavalactones, which are characteristic compounds in shell ginger. Four of these reviews described the phytochemicals of *A. zerumbet* [99,100,102,103]. However, a comprehensive review comparing the phytochemicals in the multiple medicinal parts of *A. zerumbet* which is written in English appears to be lacking.

This review summarizes the previous studies on the phytochemicals in *A. zerumbet* and clarifies the similarities and differences among the chemical constituents in multiple medicinal parts (leaves, rhizomes, fruits, seeds, and flowers) of the plant. The results contribute to the scientific validation of the traditional understanding that *A. zerumbet* possesses different medicinal properties in each part of the plant. Moreover, it provides researchers with a summary of the previous studies and directions for further studies.

## 2. Phytochemicals

### 2.1. Isolated Compounds

**Figure 1:**
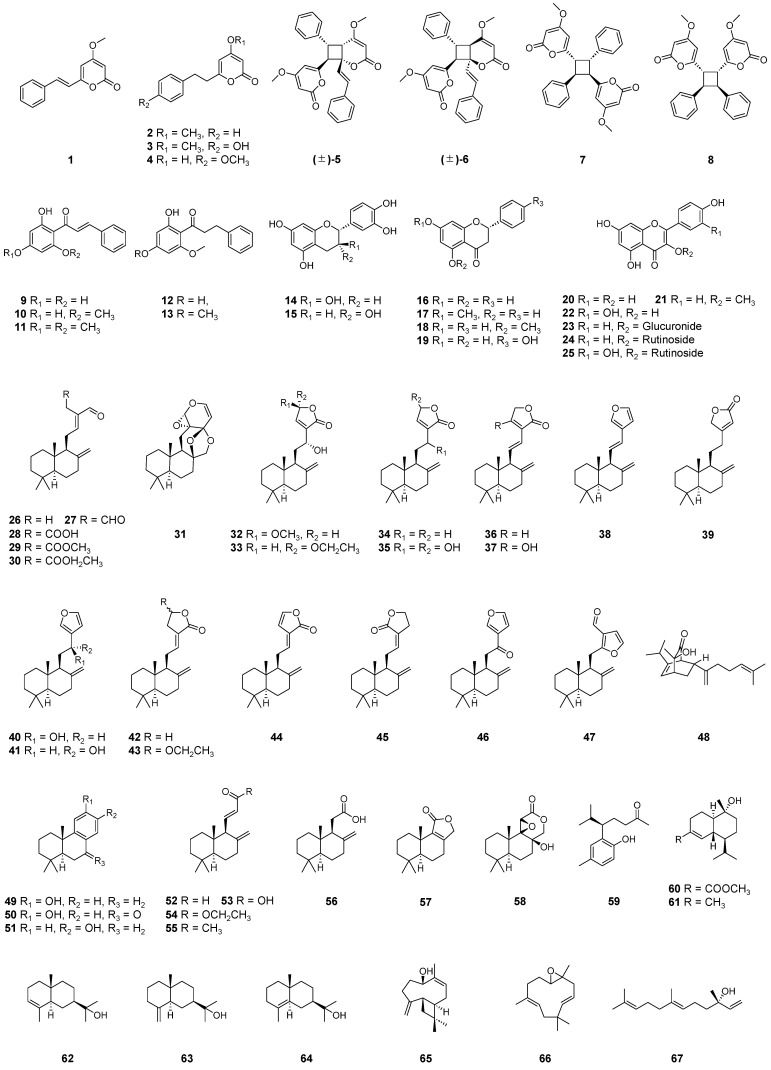
Structures of the kavalactones, chalcones, flavonoids, diterpenoids, and sesquiterpenoids isolated from *A. zerumbet*.

**Figure 2:**
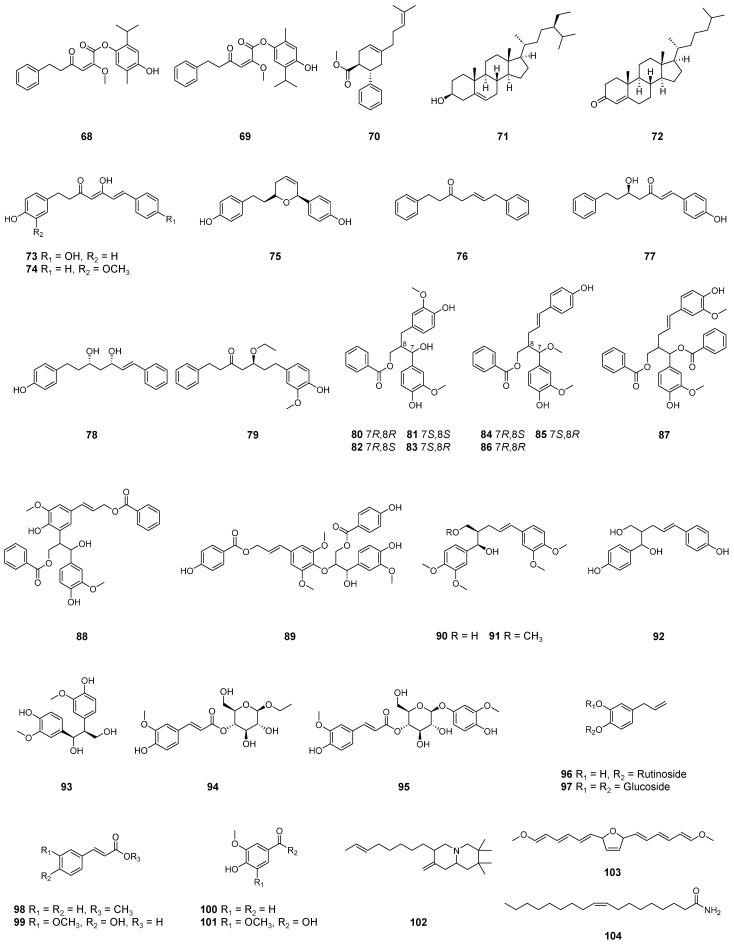
Structures of the monoterpenoids, meroterpenoids, steroids, diarylheptanoids, neolignans, glucoside esters, phenolic compounds, and other compounds isolated from *A. zerumbet*.

In total, 28 previous studies [15,19,20,28,56,59,69,78,84,93,98,104,105,106,107,108,109,110,111,112,113,114,115,116,117,118,119,120] show that more than 100 compounds have been isolated from the leaves [15,56,69,110,111,113], rhizomes [19,28,56,78,84,104,105,106,109,114,115,116,118,119,120], fruits [93], pericarps [59,107,108], seeds [20,112,116,117], and flowers [98] of *A. zerumbet* (Table 2; Figure 1 and Figure 2). Studies on the isolation of secondary metabolites from the stems and placenta were not found.

#### 2.1.1. Kavalactones

Typical kavalactones (also known as kava pyrones) are 4-methoxy-2-pyrones with styryl or phenylethyl substituents at the 6-position [121]. Kavalactones have been isolated from the leaves, rhizomes, fruits, pericarps, and flowers of *A. zerumbet*. Xuan and Teschke [122] suggested that 7,8-dihydro-5,6-dehydrokawain (**2**) was first isolated 40 years ago by Pooter et al. However, Kimura et al. [104] isolated 5,6-dehydrokawain (**1**) and 7,8-dihydro-5,6-dehydrokawain (**2**) from the rhizomes of *A. zerumbet* in 1966. Thereafter, 5,6-dehydrokawain (**1**) was isolated from the leaves [15,56,69], rhizomes [19,105,106,109], fruits [93], pericarps [59,107,108], and flowers [98] of the plant, and 7,8-dihydro-5,6-dehydrokawain (**2**) was isolated from its leaves [15,56,69,110], rhizomes [19,105,106], fruits [93], and pericarps [59,107,108].

The kavalactone derivatives **3** and **4** were isolated as new compounds from the leaves [111] and rhizomes [106] of *A. zerumbet*, respectively. Four kavalactone dimers, **5**–**8**, were isolated from the leaves [111] and pericarps [59] of the plants. (±)-Aniba dimer A (**5**) and alpingsin C (**7**) were isolated from the fruits (pericarps, placenta, and seeds) by Xiong et al. [93]. Reports on the isolation of kavalactones **1**, **2**, and **5**–**8** from the seeds are notably absent; these kavalactones have mainly been isolated from the pericarps of the fruit.

#### 2.1.2. Chalcones

Chalcones have been isolated from the rhizomes [105,109], pericarps [108], and seeds [112] of *A. zerumbet*. Pinocembrin chalcone (**9**) [109], cardamonin (**10**) [105,109], flavokawin B (**11**) [105], and dihydroflavokawin B (**13**) (a new compound) [105] have been isolated from the rhizomes.

Cardamonin (**10**) and uvangoletin (**12**) have been isolated from the pericarps [108].

From the seeds, Krishna and Chaganty [112] isolated cardamonin (**10**).

#### 2.1.3. Flavonoids

The isolations of flavonoids from the leaves [15,69,113], rhizomes [105,106,109,114], fruits [93], and seeds [112] of *A. zerumbet* have been reported. From the leaves, Mpalantinos et al. [15] isolated the five known flavonoids **14**, **15**, and **23**–**25** in 1998, and the other researchers extracted pinocembrin (**16**) [113], pinostrobin (**17**) [69], and kaempferol (**20**) [69].

Itokawa et al. [105] isolated alpinetin (**18**) from the rhizomes in 1981. Ohtsuki et al. [109] performed a phytochemical investigation of the rhizomes and isolated pinocembrin (**16**), naringenin (**19**), and 3-methoxykaempferol (**21**). Other researchers have isolated quercetin (**22**) [106] and kaempferol-3-*O*-glucuronide (**23**) [114] from the rhizomes.

Xiao et al. reported the isolation of quercetin (**22**) from the fruits [93].

Alpinetin (**18**) has been isolated from the seeds [112].

#### 2.1.4. Diterpenoids

Diterpenoids have been obtained from the rhizomes [28,56,84,106,114,115] and seeds [20,116,117] of *A. zerumbet*. Most of the isolated diterpenoids are labdane and norlabdane diterpenes. (*E*)-Labda-8(17),12-diene-15,16-dial (**27**) and (*E*)-15,16-bisnorlabda-8(17),11-dien-13-one (**55**) were isolated from the rhizomes by Itokawa et al. in 1980 [28], and compound **27** was later extracted from the rhizomes by Chompoo et al. [84], Upadhyay et al. [56], Chen et al. [106], and Taira et al. [114]. Phytochemical investigations by Xiong et al. [115] resulted in the isolation of diterpenoids **26**, **29**–**34**, **36**, **37**, **39**–**54**, and **56**–**58** from the rhizomes. Eleven of these compounds, named zerumin D1–D4 and D8–D14, were established as new compounds.

In 1995, Xu et al. [20] investigated the chemical constituents of the seeds, isolating zerumin (**37**) as a new labdane diterpene. They reported the first evidence of diterpene in the seeds [20]. Further studies by the same group afforded two new diterpenoids, zerumin A (**28**) and zerumin B (**35**), along with the two known diterpenoids **38** and **55**, from seeds [117]. Be Tu et al. [116] isolated (*E*)-labda-8(17),12-diene-15,16-dial (**27**) from the seeds.

#### 2.1.5. Sesquiterpenoids and Monoterpenoids

Two studies have reported the isolation of sesquiterpenoids from *A. zerumbet* rhizomes [115,118]. Morita et al. [118] isolated three known sesquiterpenoids, β-eudesmol (**63**), humulene epoxide II (**66**), and nerolidol (**67**), in 1996. Xiong et al. [115] isolated seven sesquiterpenoids, **59**–**65**, including two new compounds (zerumin D6 (**59**) and D5 (**60**)), in 2023.

Also in 2023, we elucidated the structures of two new monoterpene esters **68** and **69** from the pericarps of *A. zerumbet* [108].

#### 2.1.6. Meroterpenoids and Steroids

Xiong et al. [115] isolated the previously undescribed zerumin D7 (**70**) with a meroterpenoid structure from the rhizomes of *A. zerumbet* in 2023.

In 2024, Xiao et al. reported the isolation of β-sitosterol (**71**) and cholestenone (**72**) from the fruits [93].

#### 2.1.7. Diarylheptanoids and Neolignans

Diarylheptanoids **73**–**79** were isolated from the rhizomes of *A. zerumbet* by Zhang et al. [119] in 2021.

From the rhizomes of *A. zerumbet*, El-Shamy et al. [78] isolated the neolignan **92** in 2015, and Zhang et al. [119] isolated thirteen neolignans: **80**–**91** and **93**. Among them, seven compounds **80**–**86** and **88** were established as new compounds.

#### 2.1.8. Glucoside Esters, Phenolic Compounds, and Others

Masuda et al. [120] isolated two new glucoside esters **94** and **95** from the rhizomes of *A. zerumbet*.

The known phenolic compounds **96**–**98** and **99**–**101** have been isolated from the rhizomes [78,105] and fruits [93] of *A. zerumbet*, respectively.

(*E*)-2,2,3,3-Tetramethyl-8-methylene-7-(oct-6-en-1-yl)octahydro-1*H*-quinolizine (**102**) and 2,5-bis (1*E*,3*E*,5*E*)-6-methoxyhexa-1,3,5-trien-1-yl-2,5-dihydrofuran (**103**) were isolated from the seeds and rhizomes, respectively [116]. Oleamide (**104**) was isolated from the fruits [93].

### 2.2. Essential Oils

The essential oils of *A. zerumbet* mainly include monoterpenes, oxygenated monoterpenes, and oxygenated sesquiterpenes [123]. According to the previous 35 studies [2,4,8,14,18,31,34,36,37,38,41,47,49,51,52,53,54,70,74,87,92,96,97,124,125,126,127,128,129,130,131,132,133,134,135], the relative contents of the 33 major volatile components in the essential oils prepared from the leaves [2,4,8,14,31,34,36,37,38,41,47,49,51,52,53,54,70,124,125,126,127,128,129,130], rhizomes [8,14,36,70,74,130,131], fruits [53,87,92], seeds [18,38,96], and flowers [14,53,96,97,125,130,132,133] of *A. zerumbet* are shown in Table 3 and Table 4. These volatile constituents are α-thujene (930), α-pinene (939), camphene (954), sabinene (975), β-pinene (979), myrcene (990), α-phellandlene (1002), α-terpinene (1017), *p*-cymene (1024), limonene (1029), 1,8-cineole (1031), γ-terpinene (1059), *trans*-4-thujanol (1070), terpinolene (1088), linalool (1098), camphor (1146), borneol (1169), terpinen-4-ol (1177), cryptone (1185), α-terpineol (1188), cuminaldehyde (1241), (*E*)-methyl cinnamate (1378), β-caryophyllene (1419), α-humulene (1454), elemol (1549), (*E*)-nerolidol (1563), caryophyllene oxide (1583), 10-epi-γ-eudesmol (1623), γ-eudesmol (1632), β-eudesmol (1650), and α-eudesmol (1653). The numbers in parentheses are the reported Kovats Retention Index (RI) values [136].

#### 2.2.1. Leaves

The chemical constituents of the essential oils obtained from the leaves of *A. zerumbet* have been frequently reported [2,4,8,14,31,34,36,37,38,41,47,49,51,52,53,54,70,124,125,126,127,128,129,130]. In 1984, Luz et al. [124] investigated the composition of the essential oils in *A. zerumbet* leaves collected 60 km from Manausare (Brazil) and identified *p*-cymene (9.4%), 1,8-cineol (14.9%), γ-terpinene (9.5%), and 4-terpineol (20.4%) [124]. Cavalcanti et al. [41], Cunha et al. [34], de Carvalho Castro et al. [31], Gomes et al. [51], and Rocha et al. [54] clarified *p*-cymene, 1,8-cineol, and terpinen-4-ol as the main components of the essential oils from *A. zerumbet* leaves collected in Brazil. Other studies have revealed the chemical compositions of the essential oils derived from *A. zerumbet* leaves collected from Belém City, State of Pará (Brazil) in May 1997 [2], Rio de Janeiro (Brazil) [125], Núcleo de Pesquisas de Produtos Naturais (Brazil) [126], the municipality of Baturité (Brazil) in March 2010 [37], the Botanical Garden of the Universidade Federal Rural do Rio de Janeiro (GPS: 22°31′36.23 S, 44° 04′31.62 W) (Brazil) [127], Bacabal City, State of Maranhão (GPS: 4°13′2″ S, 44°46′60″ W) (Brazil) [52], and the Medicinal Plants Garden of the Federal University of Lavras (GPS: 21°12′4″ S, 44°57′58″ W) (Brazil) in September 2018 [128].

In 2009, Murakami et al. [4,47] reported a predominance of *p*-cymene, 1,8-cineole, and terpinen-4-ol in leaf essential oils prepared from *A. zerumbet* plants collected in the farms of Nihon Gettou Co., Ltd. (Okinawa, Japan). Essential oils have also been prepared from *A. zerumbet* leaves collected at the Faculty of Agriculture, University of the Ryukyus (Okinawa, Japan) [70], Nihon Gettou Co., Ltd. (Okinawa, Japan) [49], and Green Plan Shinjo Co. (Okinawa, Japan) [129].

Prudent et al. [14] identified terpinen-4-ol (29.8%) and 1,8-cineole (17.0%) in the essential oils obtained from raw *A. zerumbet* material collected in Martinique (French West Indies). They also reported (for the first time) the presence of *cis*/*trans*-sabinene hydrate, *cis*/*trans*-thujanol, *cis*/*trans*-piperitol, cuminaldehyde, (*E*)-β-farnesene, nerolidol, caryophyllene oxide, humulene oxide II, and β-, γ- and 10-epi-γ-eudesmol in leaf essential oil [14]. de Pooter et al. [36] extracted and analyzed the essential oil from *A. zerumbet* leaves collected from Orman Gardens, Giza, Egypt, in April 1990. The main ingredients were identified as sabinene (10.1%), 1,8-cineole (14.4%), γ-terpinene (11.1%), and terpinen-4-ol (17.3%) [36]. Ali et al. [8] revealed the predominance of β-pinene (10.0%), 1,8-cineole (13.2%), and terpinen-4-ol (40.9%) in the essential oil from *A. zerumbet* leaves collected in Fiji.

Padalia et al. [130,137] analyzed the essential oils from the leaves of plants cultivated at the Central Institute of Medicinal and Aromatic Plants Resource Center (India). They identified 1,8-cineole (19.2%) and terpinen-4-ol (28.4%) as the main constituents [130,137]. Ho [38] and Feng et al. [53] revealed the chemical composition of the essential oils prepared from *A. zerumbet* plants collected in Chu-Tung (Taiwan) and Guizhou Province (China), respectively. The leaves from the plants grown in Taiwan contained camphor (31.6%) [38], whereas those grown in China contained *o*-cymene (14.9%) [53].

Several studies are available on the chemical composition of essential oils extracted from *A. zerumbet* leaves collected in Brazil and Japan (Table 3). α-Thujene and α-terpinene are found in most of the essential oils in *A. zerumbet* leaves collected from Brazil, whereas camphene, camphor, (*E*)-methyl cinnamate, and α-humulene are found in most of those from Japan.

#### 2.2.2. Rhizomes

Seven studies have revealed the chemical components of the essential oils derived from *A. zerumbet* rhizomes [8,14,36,70,74,130,131]. Rhizome oils prepared from *A. zerumbet* plants collected in the Botanical Garden of the Council of Scientific and Industrial Research—Northeast Institute of Science & Technology in Jorhat (India) [131], Martinique [14], and Fiji [8] are enriched in terpinen-4-ol (46.9%, 47.3%, and 41.4%, respectively). The rhizome essential oils in plants collected from the Central Institute of Medicinal and Aromatic Plants at the Resource Center (Pantnagar, India) are dominated by 1,8-cineole (11.8%) and *endo*-fenchyl acetate (40.1%) [130], whereas those collected from *A. zerumbet* in Dehradun (Uttarakhand, India) [74] and Orman Gardens (Giza, Egypt) [36] are dominated by 1,8-cineole (11.1% and 15.9%, respectively) and terpinen-4-ol (15.4% and 20.2%, respectively). Elzaawely et al. [70] identified methyl cinnamate (15.0%) and 7,8-dihydro-5,6-dehydrokawain (21.4%) as the main constituents of rhizome oil in *A. zerumbet* plants collected from Okinawa (Japan).

#### 2.2.3. Fruits

The chemical constituents in the essential oils of *A. zerumbet* fruits have been reported by the groups of Shen et al. [88,92], Feng et al. [53], and Hou et al. [87]. The group of Shen et al. [88,92] identified 58 volatile compounds in the essential oils of *A. zerumbet* fruits collected from China. The major components were camphene (10.1%), β-pinene (15.1%), β-phellandrene (16.4%), and 1,8-cineole (11.0%) [88,92]. Feng et al. [53] identified 1,8-cineole (8.8%) in the essential oils obtained from *A. zerumbet* fruits collected in Guizhou Province (China) in July 2017. Hou et al. [87] revealed that α-pinene (7.1%), β-pinene (15.1%), and δ-cadinene (5.1%) dominate the essential oils of *A. zerumbet* fruits collected from China.

#### 2.2.4. Seeds

Three studies have reported volatile components in the essential oils prepared from *A. zerumbet* seeds [18,38,96]. Elzaawely et al. [96] prepared essential oils from the seeds of *A. zerumbet* farmed at the Faculty of Agriculture, University of the Ryukyus, Okinawa (Japan) and revealed a predominance of terpinen-4-ol (6.2%), α-terpineol (10.7%), δ-cadinene (6.2%), T-muurolol (10.8%), and α-cadinol (13.5%) in the seed oils. Lin et al. [18] analyzed the essential oils in the seeds of *A. zerumbet* plants collected from Nan-Tou County in Middle West Taiwan and identified 53 compounds dominated by sabinene (33.9%) and β-pinene (30.5%). Ho [38], who analyzed the seed oils in plants from Chu-Tung, Taiwan, identified sabinene (15.1%), (*Z*)-β-ocimene (7.9%), camphor (19.3%), and terpinen-4-ol (6.6%) as the main ingredients.

#### 2.2.5. Flowers

Eight studies have revealed the composition of flower oils from *A. zerumbet* [14,53,96,97,125,130,132,133]. The components in the oils prepared from *A. zerumbet* flowers were first clarified by Prudent et al. [14] in 1993. They showed that terpinen-4-ol (23.7%) is the major constituent in flowers collected from Martinique, French West Indies. Kerdudo et al. [97] analyzed the essential oils in flowers collected from various locations on Martinique Island in different seasons. The major volatiles were sabinene (7.0–16.5%), α-terpinene (1.9–8.1%), *p*-cymene (2.1–8.8%), 1,8-cineole (17.4–25.2%), γ-terpinene (6.9–14.7%), and terpinen-4-ol (14.7–22.6%) [97].

The flower essential oil of *A. zerumbet* collected from Pantnagar (India) contained sabinene (14.2%), 1,8-cineole (10.8%), γ-terpinene (19.4%), and terpinen-4-ol (25.1%) [133]. In another study of essential oils from *A. zerumbet* flowers collected in India, the dominant ingredients were 1,8-cineole (24.4%) and terpinen-4-ol (26.0%) [130].

Dũng et al. [132] investigated the volatile oil in flowers collected from the Xuân Phú district of Huế City, Vietnam. They identified more than 35 constituents dominated by β-pinene (34.0%). Elzaawely et al. [96] identified 1,8-cineol (16.6%), camphor (14.1%), and methyl cinnamate (12.8%) as the main components of flower oil prepared from *A. zerumbet* plants growing in Okinawa (Japan). Victório et al. [125] revealed that 1,8 cineole (15.5%), γ-terpinene (13.1%), and terpinen-4-ol (42.3%) dominate the flower oil of *A. zerumbet* in Rio de Janeiro (Brazil). The essential oils prepared from *A. zerumbet* flowers collected from China are dominated by camphene (9.5%), *m*-cymene (11.3%), and camphor (8.4%) [53].

#### 2.2.6. Stems: Aerial Parts and Others

Prudent et al. [14] first clarified the chemical constituents in essential oils prepared from *A. zerumbet* stems, revealing a predominance of terpinen-4-ol (9.6%). The essential oils in the stems of plants collected from Orman Gardens (Giza, Egypt) in April 1990 were dominated by terpinen-4-ol (16.0%) and 1,8-cineole (11.5%) [36], whereas those prepared from the stems of plants collected from China were dominated by 1,8-cineole (14.9%) [53].

Joshi et al. [133] revealed that essential oils from the aerial parts are rich in sabinene (27.8%), 1,8-cineole (17.4%), and terpinen-4-ol (14.9%). Luz et al. [134] investigated the chemical components of the essential oils in the aerial parts of *A. zerumbet* collected in the State of Piauí (GPS: 03º01′27.5″ S, 41º44′53.5″ W) (Brazil). Among 28 metabolites, the major compounds were 1,8-cineol (19.2%), γ-terpinene (14.9%), and terpinen-4-ol (27.7%) [134].

Santos et al. [135] prepared essential oils from the flowers and leaves of *A. zerumbet* collected in Aracaju City, Sergipe, Brazil (GPS: 10°55′ S, 37°03′ W). The major volatile components were *p*-cymene (10.7%), 1,8-cineole (17.6%), γ-terpinene (11.8%), and terpinen-4-ol (37.6%).

#### 2.2.7. Comparison of the Compositions of the Oils from Different Parts of *A. zerumbet*

Nine studies have reported the comparison of the compositions of the oils from different parts of *A. zerumbet* [8,14,36,38,53,70,96,125,130]. Ali et al. [8] reported similar essential oils in the leaves and rhizomes of *A. zerumbet*, with 1,8-cineole (13.2% and 28.1%, respectively) and terpinen-4-ol (40.9% and 41.4%, respectively) content. Elzaawely et al. [70] reported that 1,8-cineol (18.9%) and camphor (11.9%) dominate the leaf oil while methyl cinnamate (15.0%) and 7,8-dihydro-5,6-dehydrokawain (21.4%) dominate the rhizome oil of *A. zerumbet*.

According to Victório et al. [125], the compositions of leaf and flower oils from *A. zerumbet* are very similar and differentiated mainly by their terpinen-4-ol concentrations (19.3% in leaf oil and 42.3% in flower oil).

Ho [38] reported that camphor (31.6%), sabinene (9.4%), and γ-terpinene (8.0%) dominate the essential oils of *A. zerumbet* leaves, whereas camphor (19.3%), sabinene (15.1%), and (*Z*)-β-ocimene (7.9%) dominate the essential oils of the seeds.

Although flower oil mainly contains 1,8-cineol (16.6%), camphor (14.1%), and methyl cinnamate (12.8%), seed oil is dominated by α-terpineol (10.7%), T-muurolol (10.8%), and α-cadinol (13.5%) [96].

de Pooter et al. [36] found that the leaf, rhizome, and stem oils from *A. zerumbet* are composed mainly of sabinene (10.1%, 9.8%, and 7.5%, respectively), 1,8-cineole (14.4%, 15.9% and 11.5%, respectively), γ-terpinene (11.1%, 9.3%, and 8.2%, respectively), and terpinen-4-ol (17.3%, 20.2% and 16.0%, respectively). 

Padalia et al. [130] reported that leaf and flower oils from *A. zerumbet* are mainly represented by 1,8-cineole (19.2% and 24.4%, respectively) and terpinen-4-ol (28.4% and 26.0%, respectively), but the rhizome oil is characterized by camphene (7.8%), 1,8-cineole (11.8%), borneol (5.8%), *endo*-fenchyl acetate (40.1%), and bornyl acetate (6.9%). *endo*-Fenchyl acetate, *exo*-fenchyl acetate, and *endo*-fenchol are unique to rhizome oil [130].

Prudent et al. [14] compared the essential oils in the leaves, rhizomes, stems, and flowers of *A. zerumbet* collected from Martinique. Terpinen-4-ol was the major constituent in all parts, present at 29.8% in the leaves, 47.3% in the roots, 23.7% in the flowers, and 9.6% in the stems. 

Feng et al. [53] showed that camphene (4.4–9.5%), 1,8-cineole (7.1–14.9%), linalool (5.9–8.3%), camphor (6.2–8.4%), and borneol (4.1–8.0%) were the common major compounds in the essential oils from the stems, leaves, flowers, and fruits. The essential oils from the stems, flowers, and fruits were additionally enriched in *m*-cymene (7.1%, 11.3%, and 6.6%, respectively), whereas those from leaves were enriched in *o*-cymene (14.9%) [53].

### 2.3. Quantitative Analysis

#### 2.3.1. Kavalactones 

Table 5 lists the kavalactone contents identified in multiple medicinal parts of *A. zurumbet* [59,96,107,138,139]. Tawata et al. [138] revealed different contents of 5,6-dehydrokawain (**1**) and 7,8-dihydro-5,6-dehydrokawain (**2**) in various plant parts. More specifically, the leaves, stems, and rhizomes contain 0.01%, 0.02%, and 0.10% of compound **1**, respectively, and 0.41%, 0.08%, and 0.35% of compound **2**, respectively. Elzaawely et al. [96] first estimated the content of **2** in the fresh flowers (0.03%) and seeds (0.0003%) of *A. zerumbet* in 2007. They also reported a 0.07% proportion of **2** in fresh leaves [139]. Rao et al. [107] showed that kavalactone concentrations are higher in the pericarps (**1**: 0.21%, **2**: 0.54%) than in the leaves (**1**: 0.07%, **2**: 0.39%,) and seeds (**1**: 0.05%, **2**: 0.14%,) [107]. In 2020, we clarified the 5,6-dehydrokawain (**1**) and 7,8-dihydro-5,6-dehydrokawain (**2**) contents in the pericarps (0.16% and 0.55%, respectively), placenta (0.13% and 0.49%, respectively), and leaves (0.20% and 0.22%, respectively) of *A. zerumbet* [59]. We also found higher proportions of kavalactone dimers in the pericarps (**5**: 0.04%; **6**: 0.01%; **7**: 0.05%; **8**: 0.02%) than in the placenta (**5**: 0.01%; **6**: 0.003%; **7**: 0.01%; **8**: 0.003%) and leaves (**5**: 0.02%; **6**: 0.008%; **7**: 0.03%; **8**: 0.006%) [59].

Various studies have reported the kavalactone contents in the extracts of medicinal parts of *A. zerumbet* [67,70,75,84,93]. In the study of Chompoo et al. [84], the concentration of **1** in hexane extracts was highest in the rhizomes of *A. zerumbet* (3.13 mg/g), followed by the flowers (2.22 mg/g), stems (2.08 mg/g), leaves (1.67 mg/g), pericarps (1.58 mg/g), and seeds (0.11 mg/g). Meanwhile, the concentration of **2** in hexane extracts was highest in the flowers (6.08 mg/g), followed by the rhizomes (5.41 mg/g), stems (3.70 mg/g), leaves (3.38 mg/g), seeds (0.22 mg/g), and pericarps (0.13 mg/g) [84]. The same group also extracted kavalactones **1** and **2** in ethanol and aqueous solvents [75]. In hexane extract, Elzaawely et al. [70] obtained a higher content of **2** from fresh rhizomes (424.4 mg/g) than from fresh leaves (148.7 mg/g). Cruz et al. [67] prepared infusions from *A. zerumbet* leaves collected in Rio de Janeiro. They reported a **2** concentration of 6.63 mg/g in the extract [67]. Xiao et al. [93]. clarified kavalactones **1** and **2**, (±)-aniba dimer A (**5**), and alpingsin C (**7**) concentrations of 219.1, 301.4, 42.4, and 15.3 mg/g, respectively, in an ethyl acetate extract of the fruits.

#### 2.3.2. Diterpenoids

Chompoo et al. [84] reported that the rhizomes of *A. zerumbet* yielded the highest (*E*)-labda-8(17),12-diene-15,16-dial (**27**) contents in hexane extract (3.97 mg/g), followed by seeds (2.91 mg/g) and pericarps (0.35 mg/g). Compound **27** was absent in the hexane extracts of stems, leaves, and flowers but was present in the ethanol extract of seeds (1.00 mg/g extract) and in aqueous extracts of the rhizome, pericarp, and seeds (0.81, 0.75, and 0.96  mg/g, respectively) [75].

#### 2.3.3. Others

Victório et al. [140,141] reported the contents of kaempferol-3-*O*-glucuronide (**23**) (0.56%) and rutin (**25**) (0.15%) in the dried leaves of *A. zerumbet*.

Tavichakorntrakool et al. [79] revealed various phenolic compounds and flavonoids in *A. zerumbet* rhizomes: gallic acid (0.16 mg/g), protocatechuic acid (0.11 mg/g), *p*-hydroxybenzoic acid (0.09 mg/g), syringic acid (**101**) (0.14 mg/g), ferulic acid (**99**) (0.09 mg/g), rutin (**25**) (0.66 mg/g), myricetin (2.51 mg/g), quercetin (**22**) (9.07 mg/g), and kaempferol (**20**) (0.52 mg/g).

Xiao et al. [93] clarified the contents of **22**, **71**, **72**, **99**–**101**, and **104** in an ethyl acetate extract of the fruits.

Elzaawely et al. [96] quantitatively analyzed the phenolic compounds in fresh flowers and seeds. The flowers and seeds contained *p*-hydroxybenzoic acid (0.03 and 0.02 mg/g, respectively), syringic acid (**101**) (0.02 and 0.01 mg/g, respectively), vanillin (**100**) (0.006 and 0.01 mg/g, respectively), *p*-coumaric acid (0.005 and 0.004 mg/g, respectively), ferulic acid (**99**) (0.03 and 0.009 mg/g, respectively), and cinnamic acid (0.006 and 0.004 mg/g, respectively) [96]. 

### 2.4. Qualitative Analysis

GC–MS and LC–MS analyses of organic solvent extracts of *A. zerumbet* have been variously reported [16,25,59,60,64,70,87,95,96,139,142]. A GC–MS analysis by Kuster et al. [142] revealed the presence of kavalactones, 5,6-dehydrokawain (**1**) and 7,8-dihydro-5,6-dehydrokawain (**2**) in *A. zerumbet* leaves. da Cruz et al. [25] identified thirty-eight volatile organic compounds, seven proanthocyanidins, eleven flavonoids, and one carbohydrate in *A. zerumbet* leaves. Paulino et al. [16] clarified the volatile components in a 90% ethanol extract of the leaves. The most abundant compounds were 5,6-dehydrokawain (**1**) (8.3%), 7,8-dihydro-5,6-dehydrokawain (**2**) (19.4%), tocopherol (9.1%), β-sitosterol (**71**) (13.4%), and terpinen-4-ol (9.2%) [16]. Through an HPLC–ESI–MS/MS analysis, Ghareeb et al. [60] annotated the compounds in a methanol extract of *A. zerumbet* leaves. A total of 37 secondary metabolites were characterized as flavonoids (aglycones and glycosides) and benzoic and cinnamic acid derivatives. da Silva et al. [64] conducted an UHPLC/ESI–QTOF–MS analysis of *A. zerumbet* leaf extracts. They identified nine compounds: D-(+)-trehalose, (epi)catechin, procyanidin B2, quercetin-3-*O*-glucuronide, kaempferol-3-*O*-glucoside-3″-rhamnoside, kaempferol-3-*O*-glucuronide (**23**), isorhamnetin-3-*O*-neohesperidoside, alpinetin (**18**), and pinocembrin (**16**) in the leaves. 

Hou et al. [87] identified 24 sesquiterpenes and 15 monoterpenes in the petroleum ether extract of the fruits.

In 2020, we clarified the chemical constituents in methanol extracts of *A. zerumbet* leaves, placenta, pericarps, and seeds using LC–MS [59]. Compounds **1**, **2**, and **5**–**8** were detected in the leaf, placenta, and pericarp extracts but were absent in the seed extracts [59]. Chompoo et al. [95] identified four steroids in the leaves, two steroids in the rhizomes and seeds, eight steroids in the pericarps, three steroids in the flowers, and five steroids in the stems of *A. zerumbet*. Another GC–MS analysis by Elzaawely et al. [70,96,139] revealed the presence of phenolic compounds in the ethyl acetate extracts of leaves, rhizomes, flower, and seeds. 

## 3. Conclusions

*A. zerumbet* (Pers.) B.L.Burtt & R.M.Sm is a perennial plant widely distributed in South America, Oceania, and Asia. The medicinal properties of its leaves, rhizomes, fruits, seeds, and flowers are exploited in different clinical uses. The leaves of *A. zerumbet* have been consumed as antihypertensives and diuretics. The leaves are also believed to exhibit an anti-hemorrhoidal activity and to protect against the common cold. The rhizomes of *A. zerumbet* have been used to treat trauma, peptic ulcers, catarrh, and rheumatism. The seeds and fruits of *A. zerumbet* are traditionally used as an aromatic stomachic and cardiovascular disease treatment, respectively. *A. zerumbet* flowers have been used as a bronchitis treatment. 

This review reveals that more than 100 compounds, including kavalactones, chalcones, flavonoids, terpenoids, diarylheptanoids, and neolignans, have been isolated from the leaves, rhizomes, fruits, pericarps, seeds, and flowers of this plant. This review also summarizes the chemical components of the organic solvent extracts and essential oils obtained from the various medicinal parts of *A. zerumbet*. The major components known at present are identified as the essential oil constituents, kavalactones, and flavonoids in the leaves; the essential oil constituents, kavalactones, flavonoids, and diterpenoids in the rhizomes; the essential oil constituents, kavalactones, and diterpenoids in the fruits (placenta, pericarps, and seeds); the essential oil constituents and diterpenoids in the seeds; and the essential oil constituents in the flowers. Most of the essential oils prepared from the leaves, rhizomes, and flowers of *A. zerumbet* are dominated by 1,8-cineole and terpinen-4-ol. No volatile components were found to uniquely accumulate in each medicinal part of the plant. These results contribute to the scientific validation of the traditional understanding that *A. zerumbet* possesses different medicinal properties in each plant part.

Although the chemical constituents of *A. zerumbet* have been extensively researched, phytochemical investigations have primarily focused on the leaves, rhizomes, and flowers of this plant; the fruit parts (pericarps, placenta, and seeds) have been comparatively limited. In addition, secondary metabolites such as chalcones, diarylheptanoids, and neolignans have been isolated from shell ginger but have not been quantitatively analyzed. Therefore, phytochemical investigation of *A. zerumbet*, especially of its fruit parts (pericarps, placenta, and seeds), and comparative quantitative investigations of the phytochemicals in the multiple medicinal parts (leaves, rhizomes, fruits, seeds, and flowers) would be valuable.

## Figures and Tables

**Table 1:** Bioactivities of the extracts and essential oils of multiple medicinal parts of *A. zerumbet*.

Plant parts	Extracts	Bioactivities	References
Leaves	Essential oil	Acaricidal	[31]
		Anti-aging	[32]
		Anti-hypertensive	[33,34]
		Antimicrobial	[14,35,36,37,38]
		Anti-nociceptive	[39]
		Antioxidant	[32,40,41,42]
		Antiparasitic	[43]
		Antipsychotic	[40,44]
		Anti-schizophrenic	[45]
		Antispasmodic	[46]
		Anxiolytic-like	[47,48,49]
		Depressant	[44]
		Larvicidal	[38,50,51]
		Insecticidal	[52,53]
		Anti-melanogenic	[32]
		Molluscicidal	[51]
		Myorelaxant	[46]
		Repellent	[53]
		Tyrosinase inhibitory	[38]
		Vasorelaxant	[17,54,55]
	Aqueous extract	Antioxidant	[38]
		Antiviral	[56]
		Longevity-extending	[57]
		Singlet oxygen quenching	[58]
	Methanol extract	Anti-inflammatory	[59,60]
		Anti-nociceptive	[60]
		Antioxidant	[27,38,60,61,62]
		Antipyretic	[60]
	50% Ethanol extract	Anti-hypertensive	[63]
		Vasodilator	[63,64]
	70% Ethanol extract	Antidepressant-like	[65,66]
		Antioxidant	[66]
		Anxiolytic-like	[66]
	80% Ethanol extract	Antioxidant	[67]
	90% Ethanol extract	Cardioprotective	[16]
	Ethanol extract	Antioxidant	[68]
		Photoprotective	[68]
	Dichloromethane extracts	Antiproliferative	[69]
	Ethyl acetate extract	Antioxidant	[70]
	*n*-Hexane extract	Anti-obese	[71]
	Tea	Diuretic	[72,73]
Rhizomes	Essential oil	Antimicrobial	[36,74]
	Aqueous extract	Anti-skin disease	[75]
		Antioxidant	[75]
		Antiviral	[56]
		Hepatoprotective	[76]
	Methanol extract	Antimicrobial	[77]
		Antioxidant	[77]
		Hepatoprotective	[76,78]
	50% Ethanol extract	Antibacterial	[79]
		Cytotoxicity	[80]
	95% Ethanol extract	Anti-*Helicobacter pylori*	[81]
	Ethanol extract	Analgesic	[82]
		Anti-inflammatory	[82]
		Tyrosinase inhibitory	[83]
	*n*-Hexane extract	Antiglycation	[84]
Fruits	Essential oil	Analgesic	[85]
		Anti-atherosclerotic	[86]
		Anti-inflammatory	[85,87]
		Antimicrobial	[87]
		Endothelial protective	[7,22,88,89,90]
		Neuroprotective	[91]
		Vasodilator	[92]
	Ethyl acetate extract	Anti-hypertensive	[93]
Seeds	Essential oil	Antimicrobial	[38]
		Hypolipidemic	[18,94]
		Larvicidal	[38]
		Tyrosinase inhibitory	[38]
	Aqueous extract	Antioxidant	[38,75]
	Acetone extract	Antiatherogenic	[95]
	Methanol extract	Antioxidant	[38]
	Ethyl acetate extract	Antioxidant	[96]
	Powder	Hypolipidemic	[18,94]
Flowers	Essential oil	Antimicrobial	[97]
		Insecticidal	[97]
	Methanol extract	Anticancer	[98]
	Dichloromethane extract	Anticancer	[98]
	Ethyl acetate extract	Antioxidant	[96]

**Table 2:** Compounds isolated from the multiple medicinal parts of *A. zerumbet*.

Compounds	Source						References
Kavalactones	L	R	Fr	Pe	Se	Fl	
5,6-Dehydrokawain (**1**)	+	+	+	+	−	+	[15,19,56,59,69,93,98,104,105,106,107,108,109]
7,8-Dihydro-5,6-dehydrokawain (**2**)	+	+	+	+	−	−	[15,19,56,59,69,93,104,105,106,107,108,110]
4′-Hydroxyl dihydro-5,6-dehydrokavain (**3**)	+	−	−	−	−	−	[111]
4-Hydroxy-6-(4-methoxyphenethyl)-2*H*-pyran-2-one (**4**)	−	+	−	−	−	−	[106]
(±)-Aniba dimer A (**5**)	+	−	+	+	−	−	[59,93,110,111]
(±)-Aniba dimer C (**6**)	+	−	−	+	−	−	[59,111]
Alpingsin C (**7**)	+	−	+	+	−	−	[59,93,111]
Alpingsin D (**8**)	+	−	−	+	−	−	[59,111]
Chalcones	L	R	Fr	Pe	Se	Fl	
Pinocembrin chalcone (**9**)	−	+	−	−	−	−	[109]
Cardamonin (**10**)	−	+	−	+	+	−	[105,108,109,112]
Flavokawin B (**11**)	−	+	−	−	−	−	[105]
Uvangoletin (**12**)	−	−	−	+	−	−	[108]
Dihydroflavokawin B (**13**)	−	+	−	−	−	−	[105]
Flavonoids	L	R	Fr	Pe	Se	Fl	
(+)-Catechin (**14**)	+	−	−	−	−	−	[15]
(−)-Epicatechin (**15**)	+	−	−	−	−	−	[15]
Pinocembrin (**16**)	+	+	−	−	−	−	[109,113]
Pinostrobin (**17**)	+	−	−	−	−	−	[69]
Alpinetin (**18**)	−	+	−	−	+	−	[105,112]
Naringenin (**19**)	−	+	−	−	−	−	[109]
Kaempferol (**20**)	+	−	−	−	−	−	[69]
3-Methoxykaempferol (**21**)	−	+	−	−	−	−	[109]
Quercetin (**22**)	−	+	+	−	−	−	[93,106]
Kaempferol-3-*O*-glucuronide (**23**)	+	+	−	−	−	−	[15,114]
Kaempferol-3-*O*-rutinoside (**24**)	+	−	−	−	−	−	[15]
Rutin (**25**)	+	−	−	−	−	−	[15]
Diterpenoids	L	R	Fr	Pe	Se	Fl	
Zerumin D3 (**26**)	−	+	−	−	−	−	[115]
(*E*)-Labda-8(17),12-diene-15,16-dial (**27**)	−	+	−	−	+	−	[28,56,84,106,114,116]
Zerumin A (**28**)	−	−	−	−	+	−	[117]
Pahangensin B (**29**)	−	+	−	−	−	−	[115]
Zerumin D1 (**30**)	−	+	−	−	−	−	[115]
Zerumin D2 (**31**)	−	+	−	−	−	−	[115]
Zerumin D8 (**32**)	−	+	−	−	−	−	[115]
Zerumin D9 (**33**)	−	+	−	−	−	−	[115]
Zerumin D10 (**34**)	−	+	−	−	−	−	[115]
Zerumin B (**35**)	−	−	−	−	+	−	[117]
Villosin (**36**)	−	+	−	−	−	−	[115]
Zerumin (**37**)	−	+	−	−	+	−	[20,115]
Coronarin E (**38**)	−	−	−	−	+	−	[117]
Labda-8(17),13(14)-dien-15,16-olide (**39**)	−	+	−	−	−	−	[115]
(12*S*)-15,16-Epoxy-12-hydroxy-labda-8(17),13(16),14-triene (**40**)	−	+	−	−	−	−	[115]
(12*R*)-15,16-Epoxy-12-hydroxy-labda-8(17),13(16),14-triene (**41**)	−	+	−	−	−	−	[115]
(12*E*)-Labda-8(17),12(13)-dien-16,15-olide (**42**)	−	+	−	−	−	−	[115]
Coronarin D ethyl ether (**43**)	−	+	−	−	−	−	[115]
(12*E*)-Labda-8(17),12,14-trien-16,15-olide (**44**)	−	+	−	−	−	−	[115]
Zerumin D11 (**45**)	−	+	−	−	−	−	[115]
Zerumin D12 (**46**)	−	+	−	−	−	−	[115]
12,15-Epoxylabda-8(17),12,14-trien-16-al (**47**)	−	+	−	−	−	−	[115]
Obtunone (**48**)	−	+	−	−	−	−	[115]
Podocarpa-8,11,13-trien-12-ol (**49**)	−	+	−	−	−	−	[115]
Zerumin D13 (**50**)	−	+	−	−	−	−	[115]
Podocarpa-8,11,13-trien-13-ol (**51**)	−	+	−	−	−	−	[115]
(11*E*)-14,15,16-Trinorlabda-8(17),11-dien-13-al (**52**)	−	+	−	−	−	−	[115]
(11*E*)-14,15,16-Trinorlabda-8(17),11-dien-13-oic acid (**53**)	−	+	−	−	−	−	[115]
Zerumin D14 (**54**)	−	+	−	−	−	−	[115]
(*E*)-15,16-Bisnorlabda-8(17),11-dien-13-one (**55**)	−	+	−	−	+	−	[28,117]
13,14,15,16-Tetranorlabda-8(l7)-en-12-oic acid (**56**)	−	+	−	−	−	−	[115]
Isodrimenin (**57**)	−	+	−	−	−	−	[115]
Zerumin D4 (**58**)	−	+	−	−	−	−	[115]
Sesquiterpenoids	L	R	Fr	Pe	Se	Fl	
Zerumin D6 (**59**)	−	+	−	−	−	−	[115]
Zerumin D5 (**60**)	−	+	−	−	−	−	[115]
α-Cadinol (**61**)	−	+	−	−	−	−	[115]
α-Eudesmol (**62**)	−	+	−	−	−	−	[115]
β-Eudesmol (**63**)	−	+	−	−	−	−	[115,118]
γ-Eudesmol (**64**)	−	+	−	−	−	−	[115]
Caryophyllenol I (**65**)	−	+	−	−	−	−	[115]
(±)-Humulene epoxide II(**66**)	−	+	−	−	−	−	[118]
Nerolidol (**67**)	−	+	−	−	−	−	[118]
Monoterpenoids	L	R	Fr	Pe	Se	Fl	
2′-Methoxy-4′-oxo-6′-phenyl-2′*E*-hexenoic acid 4-hydroxy-2-isopropyl-5-methylphenyl ester (**68**)	−	−	−	+	−	−	[108]
2′-Methoxy-4′-oxo-6′-phenyl-2′*E*-hexenoic acid 4-hydroxy-5-isopropyl-2-methylphenyl ester (**69**)	−	−	−	+	−	−	[108]
Meroterpenoids	L	R	Fr	Pe	Se	Fl	
Zerumin D7 (**70**)	−	+	−	−	−	−	[115]
Steroids	L	R	Fr	Pe	Se	Fl	
*β*-Sitosterol (**71**)	−	−	+	−	−	−	[93]
Cholestenone (**72**)	−	−	+	−	−	−	[93]
Diarylheptanoids	L	R	Fr	Pe	Se	Fl	
1,2-Dihydro-bis(de-*O*-methyl)curcumin (**73**)	−	+	−	−	−	−	[119]
(4*E*,6*E*)-5-Hydroxy-1-(4-hydroxy-3-methoxyphenyl)-7-phenylhepta-4,6-dien-3-one (**74**)	−	+	−	−	−	−	[119]
(3*S*,7*S*)-5,6-Dehydro-4″-de-*O*-methylcentrolobine (**75**)	−	+	−	−	−	−	[119]
1,7-Diphenyl-5-heptene-3-one (**76**)	−	+	−	−	−	−	[119]
(−)-(*R*)-4″-Hydroxyyashabushiketol (**77**)	−	+	−	−	−	−	[119]
(3*S*,5*S*)-Alpinikatin (**78**)	−	+	−	−	−	−	[119]
5*S*-Ethoxyl-7-(4-hydroxy-3-methoxyphenyl)-1-phenyl-3-heptanone (**79**)	−	+	−	−	−	−	[119]
Neolignans	L	R	Fr	Pe	Se	Fl	
9-Benzoyloxy-3,3′-dimethoxy-8′,9′-dinor-7′,8-neoligane-4,4′,7-triol (**80**–**83**)	−	+	−	−	−	−	[119]
9-Benzoyloxy-3,7-dimethoxy-8,9′-neoligane-4,4′-diol (**84**–**86**)	−	+	−	−	−	−	[119]
1,5-Di(3′-methoxyphenyl-4′-hydroxy)-2-[(benzyloxy)methyl]-pent-4-en-1-yl benzoate (**87**)	−	+	−	−	−	−	[119]
7,8-*erythro*-3,3-Dimethoxy-9,9′-dibenzoyloxy-5′,8-neoligane-4,4′,7-triol (**88**)	−	+	−	−	−	−	[119]
Quiquelignan H (**89**)	−	+	−	−	−	−	[119]
Morinol G (**90**)	−	+	−	−	−	−	[119]
(1*R*,2*R*,4*E*)-1,5-Bis(3,4-dimethoxyphenyl)-2-(methoxymethyl)pent-4-en-1-ol (**91**)	−	+	−	−	−	−	[119]
(4*E*)-1,5-Bis(4-hydroxyphenyl)-2-(hydroxymethyl)-4-penten-1-ol (**92**)	−	+	−	−	−	−	[78]
1,2-Bis-(3-methoxy-4-hydroxyphenyl)-1,3-propanediol (**93**)	−	+	−	−	−	−	[119]
Glucoside esters	L	R	Fr	Pe	Se	Fl	
Ethyl 4-*O*-feruloyl-β-glucopyranoside (**94**)	−	+	−	−	−	−	[120]
4-Hydroxy-3-methoxyphenyl 4-*O*-feruloyl-β-glucopyranoside (**95**)	−	+	−	−	−	−	[120]
Phenolic compounds	L	R	Fr	Pe	Se	Fl	
Chavicol-β-rutinoside (**96**)	−	+	−	−	−	−	[78]
1,2,Di-O-β-D-glucopyranosyl-4-allylbenzene (**97**)	−	+	−	−	−	−	[78]
*trans*-Cinnamic acid methyl ester (**98**)	−	+	−	−	−	−	[105]
Ferulic acid (**99**)	−	−	+	−	−	−	[93]
Vanillin (**100**)	−	−	+	−	−	−	[93]
Syringic acid (**101**)	−	−	+	−	−	−	[93]
Others	L	R	Fr	Pe	Se	Fl	
(*E*)-2,2,3,3-Tetramethyl-8-methylene-7-(oct-6-en-1-yl)octahydro-1*H*-quinolizine (**102**)	−	−	−	−	+	−	[116]
2,5-Bis (1*E*,3*E*,5*E*)-6-methoxyhexa-1,3,5-trien-1-yl-2,5-dihydrofuran (**103**)	−	+	−	−	−	−	[116]
Oleamide (**104**)	−	−	+	−	−	−	[93]

Abbreviations denote the plant parts: L: leaves, R: rhizomes, Fr: fruits, Pe: pericarps, Se: seeds, and Fl: flowers.

**Table 3:** Composition of the essential oils prepared from the leaves of *A. zerumbet*.

Compounds	Relative Contents of the Essential Oils																														
	L	L	L	L	L	L	L	L	L	L	L	L	L	L	L	L	L	L	L	L	L	L	L	L	L	L	L	L	L	L	L
	BRA	BRA	BRA	BRA	BRA	BRA	BRA	BRA	BRA	BRA	BRA	BRA	BRA	BRA	JPN	JPN	JPN	JPN	JPN	JPN	JPN	JPN	JPN	JPN	JPN	JPN	JPN	JPN	MTQ	EGY	FJI
α-Thujene	+				+	+	+	+	+	+	+	+	+	+															+	+	+
α-Pinene	+	+	+		+	+	+	+	+	+	+	+	+	+	+	+	+	+	+	+	+	++	+	+		+	++	++	+	+	+
Camphene															+	+	+	+	+	+	+	+	+	+		+	+	++			
Sabinene	+	+	++		+	+	+	+	+	++	++	+++	+	+	+	+	++	+		+	+		++	+		+	+		+	++	
β-pinene	+	+	+		+	+	+	+	+	+		+		+	+	+	+	+	+	+	+	+	+	+		+	+	+	+	+	++
Myrcene			+								+									+	+	+		+			+	+		+	
α-Phellandlene																												+	+		
α-Terpinene	+	+	+		+	+	+	+		+	+	+		+															+	+	
*p*-Cymene	+	+	+		+	++	+	++	++++			+	++++	++	+++	+++	++	+++	+++	+++	+++	+++	++	+++	+	+++	++	+++	++	+	+
Limonene	+	+++	+		+	+		+	+			+	+	+	+	+	+	+	+	+	+	+	+	+		+	++	+	+		
1,8-Cineole	++		++		+++	+++	+++	+++	+++	++	++	++	+++	+++	+++	++	++	++	+++	+++	++	++	++	++	++	++	++	+	++	++	++
γ-Terpinene	+	++	++	+	++	++	++	++		++	++	++	+	++	+	+	++	+		+	+		++	+		+	+		+	++	+
*trans*-4-Thujanol			+							+		+																	+	+	
Terpinolene		+				+	+	+		+	+			+																	
Linalool		+	+			+	+	+						+	+		+	+	+	+	+	+	+	+	+	+	+		+	+	
Camphor															+	+	+	+	+	+	+	+		+	++	+	+	+		+	
Borneol																		+	+	+	+	+		+	+	+					
Terpinen-4-ol	+++	+++	++		+++	++	++++	++	+++	+++	++	+++	+++	+++	++	++	++	++	++	+	++	+	+++	+	+	++			+++	++	+++ ++
Cryptone																									+		+				
α-Terpineol	+	+	+				+			+			+	+											+				+	+	
Cuminaldehyde																		+		+	+	+		+	+	+	+				
(*E*)-Methyl cinnamate															+	+		+	+	+	+	+		+		+	+				
β-Caryophyllene	+	+	+		+	+	+	+		+	+	+		+	+	+	+	+		+	+		+	+		+		+	+	+	+
α-Humulene															+	+		+		+	+	+		+	+	+	+	+			
Elemol																															
(*E*)-Nerolidol																												+			
Caryophyllene oxide		+	+	+	+		+		+	+	+	+		+											+		+	+	+	+	+
10-Epi-γ-eudesmol																															
γ-Eudesmol																									+						
β-Eudesmol																															
α-Eudesmol																															
References	[124]	[2]	[125]	[126]	[126]	[41]	[37]	[34]	[31]	[127]	[52]	[128]	[51]	[54]	[4]	[4]	[4]	[4]	[4]	[4]	[4]	[4]	[4]	[4]	[70]	[47]	[49]	[129]	[14]	[36]	[8]

The material source is L: leaves. The plant collection regions are BRA: Brazil, JPN: Japan, MTQ: Martinique, EGY: Egypt, and FJI: Fiji. The relative contents of compounds in the essential oils are + (1.0–9.9%), ++ (10.0–19.9%), +++ (20.0–29.9%), ++++ (30.0–39.9%).

**Table 4:** Composition of the essential oils prepared from the leaves, rhizomes, fruits, seeds, and flowers of *A. zerumbet*.

Compounds	Relative Contents in the Essential Oils																											
	L	L	L	R	R	R	R	R	R	R	Fr	Fr	Fr	Se	Se	Se	Fl	Fl	Fl	Fl	Fl	Fl	Fl	Fl	Fl	Fl	Fl	Fl
	IND	TWN	CHN	IND	IND	IND	MTQ	EGY	FJI	JPN	CHN	CHN	CHN	TWN	TWN	JPN	MTQ	MTQ	MTQ	MTQ	MTQ	MTQ	VNM	JPN	BRA	IND	IND	CHN
α-Thujene	+							+										+	+	+		+			+	+		
α-Pinene	+	+	+	+				+	+		+	+	+	+	+			+	+	+		+	++		+	+		+
Camphene			+	+		+		+			++	+	+															+
Sabinene	+	+			+	+		+						++++	++			++	++	++	+	+			+	++	++	
β-pinene	+	+	+		+	+			+		++	+	++	++++	+								++++					+
Myrcene		+		+				+			+				+			+	+	+		+			+			
α-Phellandlene											+	+		+								+	+		+			
α-Terpinene						+		+										+	+	+	+	+			+			
*p*-Cymene	+				+			+	+									+	+	+	+	+					+	
Limonene	+		+	+																			+		+			
1,8-Cineole	++	+	+	++	++	++		++	+++		++	+	+		+	+		+++	+++	++	++	+++	+	++	++	++	+++	+
γ-Terpinene	+	+			+	+		+	+						+			+	+	++	+	++			++	++		
*trans*-4-Thujanol	+							+													+					+	+	
Terpinolene								+										+	+	+	+	+						
Linalool	+	+	+				+				+	+			+		+	+	+	+	+	+		+		+	+	+
Camphor		++++	+							+	+	+	+		++	+								++				+
Borneol		+	+	+							+	+				+								+				+
Terpinen-4-ol	+++	+	+	+	++	+++ ++	+++ ++	+++	+++ ++		+	+	+		+	+	+++	+++	+++	++	+++	+++		+	+++ ++	+++	+++	+
Cryptone			+							+			+			+								+				+
α-Terpineol	+	+	+	+	+	+	+	+		+	+	+	+		+	++	+	+	+	+	+	+	+	+		+	+	+
Cuminaldehyde		+																										
(*E*)-Methyl cinnamate																								++				
β-Caryophyllene			+			+	+	+	+		+	+	+	+			+	+	+	+	+	+	++		+		+	+
α-Humulene		+														+	+						+					
Elemol					+							+				+	+											+
(*E*)-Nerolidol		+	+				+					+			+		+			+	+		+					+
Caryophyllene oxide		+	+				+		+		+	+	+		+		+		+	+	+		+			+		+
10-Epi-γ-eudesmol							+										+											
γ-Eudesmol					+											+	+							+				
β-Eudesmol			+				+					+					+											+
α-Eudesmol					+												+											
References	[130]	[38]	[53]	[130]	[74]	[131]	[14]	[36]	[8]	[70]	[92]	[53]	[87]	[18]	[38]	[96]	[14]	[97]	[97]	[97]	[97]	[97]	[132]	[96]	[125]	[133]	[130]	[53]

The material sources are L: leaves, R: rhizomes, Fr: fruits, Se: seeds, and Fl: flowers. The plant collection regions are IND: India, TWN: Taiwan, CHN: China, MTQ: Martinique, EGY: Egypt, FJI: Fiji, JPN: Japan, VNM: Vietnam, and BRA: Brazil. The relative contents of compounds in the essential oils are + (1.0–9.9%), ++ (10.0–19.9%), +++ (20.0–29.9%), ++++ (30.0–39.9%).

**Table 5:** Contents of major kavalactones in multiple medicinal parts of *A. zerumbet*.

Compounds	Plant Parts	Solvent	Content (%)	References
5,6-Dehydrokawain (**1**)	Fresh leaves	Ethanol	0.01	[138]
	Dried leaves	*n*-Hexane	0.07	[107]
		Methanol	0.20	[59]
	Fresh rhizomes	Ethanol	0.10	[138]
	Dried pericarps	*n*-Hexane	0.21	[107]
		Methanol	0.16	[59]
	Dried seeds	*n*-Hexane	0.05	[107]
	Dried placenta	Methanol	0.13	[59]
	Fresh stems	Ethanol	0.02	[138]
7,8-Dihydro-5,6-dehydrokawain (**2**)	Fresh leaves	Ethanol	0.41	[138]
		Water	0.07	[139]
	Dried leaves	*n*-Hexane	0.39	[107]
		Methanol	0.22	[59]
	Fresh rhizomes	Ethanol	0.35	[138]
	Dried pericarps	*n*-Hexane	0.54	[107]
		Methanol	0.55	[59]
	Fresh seeds	Water	0.0003	[96]
	Dried seeds	*n*-Hexane	0.14	[107]
	Fresh flowers	Water	0.03	[96]
	Dried placenta	Methanol	0.49	[59]
	Fresh stems	Ethanol	0.08	[138]

## References

[1] Lim T.K. (2016). Alpinia Zerumbet.

[2] Zoghbi M.D.G.B., Andrade E.H.A., Maia J.G.S. (1999). Volatile Constituents from Leaves and Flowers of *Alpinia speciosa* K. Schum. and *A. purpurata* (Viell.) Schum. Flavour Fragr. J..

[3] Batista T.S.C., Barros G.S., Damasceno F.C., Cândido E.A.F., Batista M.V.A. (2021). Chemical Characterization and Effects of Volatile Oil of *Alpinia zerumbet* on the Quality of Collagen Deposition and Caveolin-1 Expression in a Muscular Fibrosis Murine Model. Braz. J. Biol..

[4] Murakami S., Li W., Matsuura M., Satou T., Hayashi S., Koike K. (2009). Composition and Seasonal Variation of Essential Oil in *Alpinia zerumbet* from Okinawa Island. J. Nat. Med..

[5] Gomes P.W.P., Martins L., Gomes E., Muribeca A., Pamplona S., Komesu A., Bichara C., Rai M., Silva C., Silva M. (2022). Antiviral Plants from Marajó Island, Brazilian Amazon: A Narrative Review. Molecules.

[6] Victório C.P. (2011). Therapeutic Value of the Genus *Alpinia*, Zingiberaceae. Rev. Bras. Farmacogn..

[7] Shen X.C., Tao L., Li W.K., Zhang Y.Y., Luo H., Xia Y.Y. (2012). Evidence-Based Antioxidant Activity of the Essential Oil from Fructus A. Zerumbet on Cultured Human Umbilical Vein Endothelial Cells’ Injury Induced by Ox-LDL. BMC Complement. Altern. Med..

[8] Ali S., Sotheeswaran S., Tuiwawa M., Smith R.M. (2002). Comparison of the Composition of the Essential Oils of *Alpinia* and *Hedychium* Species—Essential Oils of Fijian Plants, Part 1. J. Essent. Oil Res..

[9] Agrawal N.K. (2015). Physico-Chemical and Natural Product Investigations of Essential Oil and Variously Extracted Medicinally Useful Materials From the Rhizomes of *Alpinia speciosa* K. Schum. Int. J. Pharmacogn..

[10] Nadkarni K.M. (1908). Indian Materia Medica Vol. I..

[11] Kuraya E., Miyafuji Y., Takemoto A., Itoh S. (2014). The Effect of Underwater Shock Waves on Steam Distillation of *Alpinia zerumbet* Leaves. Trans. Mat. Res. Soc. Jpn..

[12] Longuefosse J.L., Nossin E. (1996). Medical Ethnobotany Survey in Martinique. J. Ethnopharmacol..

[13] Hanh N.P., Binh N.Q., Adhikari B.S. (2014). Distribution of Alpinia (*Zingiberaceae*) and Their Use Pattern in Vietnam. J. Biodivers. Endanger. Species.

[14] Prudent D., Perineau F., Bessiere J.M., Michel G., Bravo R. (1993). Chemical Analysis, Bacteriostatic and Fungistatic Properties of the Essential Oil of the Atoumau from Martinique (*Alpinia speciosa* K. Schum.). J. Essent. Oil Res..

[15] Mpalantinos M.A., de Moura R.S., Parente J.P., Kuster R.M. (1998). Biologically Active Flavonoids and Kava Pyrones from the Aqueous Extract of *Alpinia zerumbet*. Phytother. Res..

[16] Paulino E.T., Barros Ferreira A.K., da Silva J.C.G., Ferreira Costa C.D., Smaniotto S., de Araújo-Júnior J.X., Silva Júnior E.F., Bortoluzzi J.H., Nogueira Ribeiro Ê.A. (2019). Cardioprotective Effects Induced by Hydroalcoholic Extract of Leaves of *Alpinia zerumbet* on Myocardial Infarction in Rats. J. Ethnopharmacol..

[17] Lahlou S., Galindo C.A.B., Leal-Cardoso J.H., Fonteles M.C., Duarte G.P. (2002). Cardiovascular Effects of the Essential Oil of *Alpinia zerumbet* Leaves and Its Main Constituent, Terpinen-4-Ol, in Rats: Role of the Autonomic Nervous System. Planta Med..

[18] Lin L.Y., Peng C.C., Liang Y.J., Yeh W.T., Wang H.E., Yu T.H., Peng R.Y. (2008). *Alpinia zerumbet* Potentially Elevates High-Density Lipoprotein Cholesterol Level in Hamsters. J. Agric. Food Chem..

[19] Hsu S.Y., Lin M.H., Lin L.C., Chou C.J. (1994). Toxicologic Studies of Dihydro-5,6-Dihydrokawain and 5,6-Dehydrokawain. Planta Med..

[20] Xu H.X., Hui D., Sim K.Y. (1995). The Isolation of a New Labdane Diterpene from the Seeds of *Alpinia zerumbet*. Nat. Prod. Lett..

[21] Liao J., Fu L., Tai S., Xu Y., Wang S., Guo L., Guo D., Du Y., He J., Yang H. (2024). Essential Oil from Fructus *Alpiniae zerumbet* Ameliorates Vascular Endothelial Cell Senescence in Diabetes by Regulating PPAR-γ Signalling: A 4D Label-Free Quantitative Proteomics and Network Pharmacology Study. J. Ethnopharmacol..

[22] Ji Y.P., Shi T.Y., Zhang Y.Y., Lin D., Linghu K.G., Xu Y.N., Tao L., Lu Q., Shen X.C. (2019). Essential Oil from Fructus Alpinia Zerumbet (Fruit of *Alpinia zerumbet* (Pers.) Burtt.et Smith) Protected against Aortic Endothelial Cell Injury and Inflammation in Vitro and in Vivo. J. Ethnopharmacol..

[23] Kirtikar K.R., Basu B.D. (1936). Indian Medicinal Plants Vol. IV.

[24] Tawata S., Fukuta M., Xuan T.D., Deba F. (2008). Total Utilization of Tropical Plants *Leucaena Leucocephala* and *Alpinia zerumbet*. J. Pestic. Sci..

[25] da Cruz J.D., Mpalantinos M.A., de Oliveira L.R., Branches T.G., Xavier A., Souza F.D.C.D.A., Aguiar J.P.L., Ferreira J.L.P., de Andrade Silva J.R., Amaral A.C.F. (2023). Nutritional and Chemical Composition of *Alpinia zerumbet* Leaves, a Traditional Functional Food. Food Res. Int..

[26] Okazaki K., Sumitani H., Takahashi K., Isegawa Y. (2023). Mode of Antifungal Action of Daito-*Gettou* (*Alpinia zerumbet* var. exelsa) Essential Oil against Aspergillus Brasiliensis. Foods.

[27] Chan E.W.C., Lim Y.Y., Wong L.F., Lianto F.S., Wong S.K., Lim K.K., Joe C.E., Lim T.Y. (2008). Antioxidant and Tyrosinase Inhibition Properties of Leaves and Rhizomes of Ginger Species. Food Chem..

[28] Itokawa H., Morita M., Mihashi S. (1980). Labdane and Bisnorlabdane Type Diterpenes from *Alpinia speciosa* K. SCHUM. Chem. Pharm. Bull..

[29] Kimura Y. (1939). Pharmacognostic Study on the Seeds of Species of the Genus *Alpinia* Native to Japan. Yakugaku Zasshi.

[30] Chen T.L., Chen P.W., Tsung T.T. Taiwan Aboriginal Traditional *Alpinia zerumbet* Handicraft Preparation Study. Proceedings of the International Association of Societies for Design Research (IASDR).

[31] de Carvalho Castro K.N., Canuto K.M., de Sousa Brito E., Costa-Júnior L.M., de Andrade I.M., Magalhães J.A., Barros D.M.A. (2018). *In Vitro* Efficacy of Essential Oils with Different Concentrations of 1,8-Cineole against *Rhipicephalus* (*Boophilus*) *microplus*. Rev. Bras. Parasitol. Vet..

[32] Tu P.T.B., Tawata S. (2015). Anti-Oxidant, Anti-Aging, and Anti-Melanogenic Properties of the Essential Oils from Two Varieties of *Alpinia zerumbet*. Molecules.

[33] Lahlou S., Leal Interaminense L.F., Leal-Cardoso J.H., Duarte G.P. (2003). Antihypertensive Effects of the Essential Oil of *Alpinia zerumbet* and Its Main Constituent, Terpinen-4-Ol, in DOCA-Salt Hypertensive Conscious Rats. Fundam. Clin. Pharmacol..

[34] Cunha G.H., Fechine F.V., Frota Bezerra F.A., Moraes M.O., Silveira E.R., Canuto K.M., Moraes M.E.A. (2016). Comparative Study of the Antihypertensive Effects of Hexane, Chloroform and Methanol Fractions of Essential Oil of *Alpinia zerumbet* in Rats Wistar. Rev. Bras. Plantas Med..

[35] Victório C.P., Alviano D.S., Alviano C.S., Lage C.L.S. (2009). Chemical Composition of the Fractions of Leaf Oil of *Alpinia zerumbet* (Pers.) B.L. Burtt & R.M. Sm. and Antimicrobial Activity. Rev. Bras. Farmacogn..

[36] de Pooter H.L., Aboutabl E.A., El-Shabrawy A.O. (1995). Chemical Composition and Antimicrobial Activity of Essential Oil of Leaf, Stem and Rhizome of *Alpinia speciosa* (J.C.Wendl.) K.Schum. Grown in Egypt. Flavour Fragr. J..

[37] Mendes F.R.S., Silva F.G.E., Sousa E.O., Rodrigues F.F.G., Costa J.G.M., Monte F.J.Q., Lemos T.L.G., Assunção J.C.C. (2015). Essential Oil of *Alpinia zerumbet* (Pers.) B.L. Burtt. & R.M. Sm. (Zingiberaceae): Chemical Composition and Modulation of the Activity of Aminoglycoside Antibiotics. J. Essent. Oil Res..

[38] Ho J.C. (2010). Chemical Composition and Bioactivity of Essential Oil of Seed and Leaf from *Alpinia speciosa* Grown in Taiwan. J. Chin. Chem. Soc..

[39] de Araújo Pinho F.V.S., Coelho-De-Souza A.N., Morais S.M., Ferreira Santos C., Leal-Cardoso J.H. (2005). Antinociceptive Effects of the Essential Oil of *Alpinia zerumbet* on Mice. Phytomedicine.

[40] de Araújo F.Y.R., de Oliveira G.V., Gomes P.X.L., Soares M.A., Silva M.I.G., Carvalho A.F., de Moraes M.O., de Moraes M.E.A., Vasconcelos S.M.M., Viana G.S.B. (2011). Inhibition of Ketamine-Induced Hyperlocomotion in Mice by the Essential Oil of *Alpinia zerumbet*: Possible Involvement of an Antioxidant Effect. J. Pharm. Pharmacol..

[41] Cavalcanti B.C., Ferreira J.R.O., Cabral I.O., Magalhães H.I.F., de Oliveira C.C., Rodrigues F.A.R., Rocha D.D., Barros F.W.A., da Silva C.R., Júnior H.V.N. (2012). Genetic Toxicology Evaluation of Essential Oil of *Alpinia zerumbet* and Its Chemoprotective Effects against H2O2-Induced DNA Damage in Cultured Human Leukocytes. Food Chem. Toxicol..

[42] Kuraya E., Yamashiro R., Touyama A., Nakada S., Watanabe K., Iguchi A., Itoh S. (2017). Aroma Profile and Antioxidant Activity of Essential Oil from *Alpinia zerumbet*. Nat. Prod. Commun..

[43] Pereira P.S., Maia A.J., Duarte A.E., Oliveira-Tintino C.D.M., Tintino S.R., Barros L.M., Vega-Gomez M.C., Rolón M., Coronel C., Coutinho H.D.M. (2018). Cytotoxic and Anti-Kinetoplastid Potential of the Essential Oil of *Alpinia speciosa* K. Schum. Food Chem. Toxicol..

[44] de Araújo F.Y.R., Silva M.I.G., Moura B.A., de Oliveira G.V., Leal L.A.K.M., Vasconcelos S.M.M., Viana G.S.B., de Moraes M.O., de Sousa F.C.F., Macêdo D.S. (2010). Central Nervous System Effects of the Essential Oil of the Leaves of *Alpinia zerumbet* in Mice. J. Pharm. Pharmacol..

[45] de Araújo F.Y.R., Chaves Filho A.J.M., Nunes A.M., de Oliveira G.V., Gomes P.X.L., Vasconcelos G.S., Carletti J., de Moraes M.O., de Moraes M.E., Vasconcelos S.M.M. (2021). Involvement of Anti-Inflammatory, Antioxidant, and BDNF up-Regulating Properties in the Antipsychotic-like Effect of the Essential Oil of *Alpinia zerumbet* in Mice: A Comparative Study with Olanzapine. Metab. Brain Dis..

[46] Bezerra M.A.C., Leal-Cardoso J.H., Coelho-De-Souza A.N., Criddle D.N., Fonteles M.C. (2000). Myorelaxant and Antispasmodic Effects of the Essential Oil of *Alpinia speciosa* on Rat Ileum. Phytother. Res..

[47] Murakami S., Matsuura M., Satou T., Hayashi S., Koike K. (2009). Effects of the Essential Oil from Leaves of *Alpinia zerumbet* on Behavioral Alterations in Mice. Nat. Prod. Commun..

[48] Satou T., Murakami S., Matsuura M., Hayashi S., Koike K. (2010). Anxiolytic Effect and Tissue Distribution of Inhaled *Alpinia zerumbet* Essential Oil in Mice. Nat. Prod. Commun..

[49] Satou T., Kasuya H., Takahashi M., Murakami S., Hayashi S., Sadamoto K., Koike K. (2011). Relationship between Duration of Exposure and Anxiolytic-like Effects of Essential Oil from *Alpinia zerumbet*. Flavour Fragr. J..

[50] Freitas F.P., Freitas S.P., Lemos G.C.S., Vieira I.J.C., Gravina G.A., Lemos F.J.A. (2010). Comparative Larvicidal Activity of Essential Oils from Three Medicinal Plants against *Aedes aegypti* L.. Chem. Biodivers..

[51] Gomes P.R.B., Everton G.O., Fontenele M.A., Souza R.D., de Freitas A.C., Lima Hunaldo V.K., Louzeiro H.C., Rodrigues N.F.M., Reis J.B., Filho V.E.M. (2023). Chemical Composition, Larvicidal and Molluscicidal Activity of the Essential Oil *Alpinia zerumbet*. J. Essent. Oil-Bear Plants.

[52] de Silva Barbosa D.R., dos Santos R.B.V., Santos F.M.P., da Silva Junior P.J., de Oliveira Neto F.M., Silva G.N., de Andrade Dutra K., do Amaral Ferraz Navarro D.M. (2022). Evaluation of *Cymbopogon flexuosus* and *Alpinia zerumbet* Essential Oils as Biopesticides against *Callosobruchus maculatus*. J. Plant Dis. Prot..

[53] Feng Y.X., Zhang X., Wang Y., Chen Z.Y., Lu X.X., Du Y.S., Du S.S. (2021). The Potential Contribution of Cymene Isomers to Insecticidal and Repellent Activities of the Essential Oil from *Alpinia zerumbet*. Int. Biodeterior. Biodegrad..

[54] Rocha D.G., Holanda T.M., Braz H.L.B., de Moraes J.A.S., Marinho A.D., Maia P.H.F., de Moraes M.E.A., Fechine-Jamacaru F.V., de Moraes Filho M.O. (2023). Vasorelaxant Effect of *Alpinia zerumbet’s* Essential Oil on Rat Resistance Artery Involves Blocking of Calcium Mobilization. Fitoterapia.

[55] Pinto N.V., Assreuy A.M.S., Coelho-de-Souza A.N., Ceccatto V.M., Magalhães P.J.C., Lahlou S., Leal-Cardoso J.H. (2009). Endothelium-Dependent Vasorelaxant Effects of the Essential Oil from Aerial Parts of *Alpinia zerumbet* and Its Main Constituent 1,8-Cineole in Rats. Phytomedicine.

[56] Upadhyay A., Chompoo J., Kishimoto W., Makise T., Tawata S. (2011). HIV-1 Integrase and Neuraminidase Inhibitors from *Alpinia zerumbet*. J. Agric. Food Chem..

[57] Upadhyay A., Chompoo J., Taira N., Fukuta M., Tawata S. (2013). Significant Longevity-Extending Effects of *Alpinia zerumbet* Leaf Extract on the Life Span of *Caenorhabditis elegans*. Biosci. Biotechnol. Biochem..

[58] Liao M.C., Arakaki H., Li Y., Takamiyagi A., Tawata S., Aniya Y., Sakurai H., Nonaka S. (2000). Inhibitory Effects of *Alpinia speciosa* K. SCHUM on the Porphyrin Photooxidative Reaction. J. Dermatol..

[59] Nishidono Y., Okada R., Iwama Y., Okuyama T., Nishizawa M., Tanaka K. (2020). Anti-Inflammatory Kavalactones from *Alpinia zerumbet*. Fitoterapia.

[60] Ghareeb M.A., Sobeh M., Rezq S., El-Shazly A.M., Mahmoud M.F., Wink M. (2018). HPLC-ESI-MS/MS Profiling of Polyphenolics of a Leaf Extract from *Alpinia zerumbet* (Zingiberaceae) and Its Anti-Inflammatory, Anti-Nociceptive, and Antipyretic Activities In Vivo. Molecules.

[61] Wong L.F., Lim Y.Y., Omar M. (2009). Antioxidant and Antimicrobal Activities of Some *Alpinia* Species. J. Food Biochem..

[62] Chan E.W.C., Lim Y.Y., Wong S.K., Lim K.K., Tan S.P., Lianto F.S., Yong M.Y. (2009). Effects of Different Drying Methods on the Antioxidant Properties of Leaves and Tea of Ginger Species. Food Chem..

[63] de Moura R.S., Emiliano A.F., de Carvalho L.C.R.M., Souza M.A.V., Guedes D.C., Tano T., Resende A.C. (2005). Antihypertensive and Endothelium-Dependent Vasodilator Effects of *Alpinia zerumbet*, a Medicinal Plant. J. Cardiovasc. Pharmacol..

[64] da Silva M.A., de Carvalho L.C.R.M., Victório C.P., Ognibene D.T., Resende A.C., de Souza M.A.V. (2021). Chemical Composition and Vasodilator Activity of Different *Alpinia zerumbet* Leaf Extracts, a Potential Source of Bioactive Flavonoids. Med. Chem. Res..

[65] Bevilaqua F., Mocelin R., Grimm C., da Silva Junior N.S., Buzetto T.L.B., Conterato G.M.M., Roman W.A., Piato A.L. (2016). Involvement of the Catecholaminergic System on the Antidepressant-like Effects of *Alpinia zerumbet* in Mice. Pharm. Biol..

[66] Roman Junior W.A., Piato A.L., Marafiga Conterato G.M., Wildner S.M., Marcon M., Moreira S., Santo G.D., Mocelin R., Emanuelli T., de Moraes Santos C.A. (2013). Psychopharmacological and Antioxidant Effects of Hydroethanolic Extract of *Alpinia zerumbet* Leaves in Mice. Pharmacogn. J..

[67] da Cruz J.D., Mpalantinos M.A., Ramos A.D.S., Ferreira J.L.P., de Oliveira A.A., Júnior N.L.N., Silva J.R.D.A., Amaral A.C.F. (2020). Chemical Standardization, Antioxidant Activity and Phenolic Contents of Cultivated *Alpinia zerumbet* Preparations. Ind. Crops Prod..

[68] Lima R.M., Polonini H.C., de Souza K.C., Brandão M.A.F., Salgado I., Raposo N.R.B. (2015). Assessment of Different Biological Capacities of *Alpinia speciosa* (Pers.) B.L. Burtt and R.M. Sm. J. Young Pharm..

[69] Junior W.A.R., Gomes D.B., Zanchet B., Schönell A.P., Diel K.A.P., Banzato T.P., Ruiz A.L.T.G., Carvalho J.E., Neppel A., Barison A. (2017). Antiproliferative Effects of Pinostrobin and 5,6-Dehydrokavain Isolated from Leaves of *Alpinia zerumbet*. Rev. Bras. Farmacogn..

[70] Elzaawely A.A., Xuan T.D., Tawata S. (2007). Essential Oils, Kava Pyrones and Phenolic Compounds from Leaves and Rhizomes of *Alpinia zerumbet* (Pers.) B.L. Burtt. & R.M. Sm. and Their Antioxidant Activity. Food Chem..

[71] Niwano Y., Beppu F., Shimada T., Kyan R., Yasura K., Tamaki M., Nishino M., Midorikawa Y., Hamada H. (2009). Extensive Screening for Plant Foodstuffs in Okinawa, Japan with Anti-Obese Activity on Adipocytes in Vitro. Plant Foods Hum. Nutr..

[72] Laranja S.M., Bergamaschi C.M., Schor N. (1991). Evaluation of Acute Administration of Natural Products with Potential Diuretic Effects, in Humans. Mem. Inst. Oswaldo Cruz..

[73] Laranja S.M.R., Bergamaschi C.M., Schor N. (1992). Evaluation of Three Plants with Potential Diuretic Effect. Rev. Assoc. Med. Bras..

[74] Indrayan A.K., Tyagi P.K., Agrawal N.K. (2010). Chemical Composition and Antimicrobial Activity of the Essential Oil of *Alpinia Speciosa* K. Schum. Rhizome From India. J. Essent. Oil Res..

[75] Chompoo J., Upadhyay A., Fukuta M., Tawata S. (2012). Effect of *Alpinia zerumbet* Components on Antioxidant and Skin Diseases-Related Enzymes. BMC Complement. Altern. Med..

[76] El-Hawary S., Kassem H., Motaal A.A., Tawfik W., Hassanein H., El-Shamy S. (2013). GC-MS Analysis of the Essential Oil of *Alpinia zerumbet* (Pers.) B.L. and in Vitro Hepatoprotection and Cytotoxicity Study. MPC-4. Planta Med..

[77] Chen I.N., Chang C.C., Ng C.C., Wang C.Y., Shyu Y.T., Chang T.L. (2008). Antioxidant and Antimicrobial Activity of Zingiberaceae Plants in Taiwan. Plant Foods Hum. Nutr..

[78] Hammouda F.M., El-Hawary S.S., Kassem H.A., Motaal A.A.A., Nazif N.M., El-Shamy S.S. (2015). Hepatoprotective and Antioxidant Activities of Phenolic Compounds Isolated from *Alpinia zerumbet* (Pers.) B.L. Grown in Egypt. Res. J. Pharm. Biol. Chem. Sci..

[79] Tavichakorntrakool R., Lulitanond A., Sangka A., Sungkeeree S., Weerapreeyakul N. (2019). Antibacterial Activity and Bioactive Compounds of 50% Hydroethanolic Extract of *Alpinia zerumbet* (Pers.) B.L. Burtt & R.M. Sm. Asian Pac. J. Trop. Biomed..

[80] Machana S., Weerapreeyakul N., Barusrux S., Nonpunya A., Sripanidkulchai B., Thitimetharoch T. (2011). Cytotoxic and Apoptotic Effects of Six Herbal Plants against the Human Hepatocarcinoma (HepG2) Cell Line. Chin. Med..

[81] Wang Y.C., Huang T.L. (2005). Screening of Anti-*Helicobacter pylori* Herbs Deriving from Taiwanese Folk Medicinal Plants. FEMS Immunol. Med. Microbiol..

[82] Thenmozhi S., Sureshkumar S., Rajesh V. (2011). Evaluation of Analgesic and Anti-Inflammatory Activity of *Alpinia speciosa* K. Schum Rhizomes. J. Pharm. Res..

[83] Masuda T., Fujita N., Odaka Y., Takeda Y., Yonemori S., Nakamoto K., Kuninaga H. (2007). Tyrosinase Inhibitory Activity of Ethanol Extracts from Medicinal and Edible Plants Cultivated in Okinawa and Identification of a Water-Soluble Inhibitor from the Leaves of *Nandina Domestica*. Biosci. Biotechnol. Biochem..

[84] Chompoo J., Upadhyay A., Kishimoto W., Makise T., Tawata S. (2011). Advanced Glycation End Products Inhibitors from *Alpinia zerumbet* Rhizomes. Food Chem..

[85] Xiao R.Y., Wu L.J., Hong X.X., Tao L., Luo P., Shen X.C. (2018). Screening of Analgesic and Anti-Inflammatory Active Component in Fructus *Alpiniae zerumbet* Based on Spectrum–Effect Relationship and GC–MS. Biomed. Chromatogr..

[86] Wang S.Q., Xiang J., Zhang G.Q., Fu L.Y., Xu Y.N., Chen Y., Tao L., Hu X.X., Shen X.C. (2024). Essential Oil from Fructus *Alpinia zerumbet* Ameliorates Atherosclerosis by Activating PPARγ-LXRα-ABCA1/G1 Signaling Pathway. Phytomedicine.

[87] Hou J., Gong H., Gong Z., Qin X., Nie J., Zhu H., Zhong S. (2023). Chemical Composition and Potential Antimicrobial and Anti-Inflammatory Activities of Essential Oil from Fruits of *Alpinia zerumbet* (Pers.) B.L.Burtt & R.M.Sm. Chem. Biodivers..

[88] Xiao T., Zeng Y., Xu Y., Zhang Y., Jiang Y., Tao L., Shen X. (2014). The Endothelial Protective Properties of Essential Oil from Fructus Alpiniae Zerumbet via the Akt/NOS-NO Signaling Pathway In Vitro. Planta Med..

[89] Zhang Y., Zhao S., Tu M., He L., Xu Y., Gan S., Shen X. (2022). Inhibitory Effect of Essential Oil from Fructus of *Alpinia zerumbet* on Endothelial-to-Mesenchymal Transformation Induced by TGF-β 1and Downregulation of KLF4. J. Cardiovasc. Pharmacol..

[90] Zhang Y., Li C., Huang Y., Zhao S., Xu Y., Chen Y., Jiang F., Tao L., Shen X. (2020). EOFAZ Inhibits Endothelial-to-Mesenchymal Transition through Downregulation of KLF4. Int. J. Mol. Med..

[91] Yang H., Gan S., Jiang Z., Song X., Chen T., Xu Y., Fu L., Zhang Y., Tao L., Shen X. (2020). Protective Effects of Essential Oil from Fructus *Alpiniae zerumbet* on Retinal Müller Gliosis via the PPAR-γ-p-CREB Signaling Pathway. Chin. Med..

[92] Tao L., Hu H.S., Shen X.C. (2013). Endothelium-Dependent Vasodilatation Effects of the Essential Oil from Fructus Alpiniae Zerumbet (EOFAZ) on Rat Thoracic Aortic Rings *in Vitro*. Phytomedicine.

[93] Xiao T., Wu A., Wang X., Guo Z., Huang F., Cheng X., Shen X., Tao L. (2024). Anti-Hypertensive and Composition as Well as Pharmacokinetics and Tissues Distribution of Active Ingredients from *Alpinia zerumbet*. Fitoterapia.

[94] Chuang C.M., Wang H.E., Peng C.C., Chen K.C., Peng R.Y. (2011). Hypolipidemic Effects of Different Angiocarp Parts of *Alpinia zerumbet*. Pharm. Biol..

[95] Chompoo J., Upadhyay A., Gima S., Fukuta M., Tawata S. (2012). Antiatherogenic Properties of Acetone Extract of *Alpinia zerumbet* Seeds. Molecules.

[96] Elzaawely A.A., Xuan T.D., Koyama H., Tawata S. (2007). Antioxidant Activity and Contents of Essential Oil and Phenolic Compounds in Flowers and Seeds of *Alpinia zerumbet* (Pers.) B.L. Burtt. & R.M. Sm. Food Chem..

[97] Kerdudo A., Ellong E.N., Burger P., Gonnot V., Boyer L., Chandre F., Adenet S., Rochefort K., Michel T., Fernandez X. (2017). Chemical Composition, Antimicrobial and Insecticidal Activities of Flowers Essential Oils of *Alpinia zerumbet* (Pers.) B.L.Burtt & R.M.Sm. from Martinique Island. Chem. Biodivers..

[98] Zahra M.H., Salem T.A.R., El-Aarag B., Yosri N., EL-Ghlban S., Zaki K., Marei A.H., El-Wahed A.A., Saeed A., Khatib A. (2019). *Alpinia zerumbet* (Pers.): Food and Medicinal Plant with Potential In Vitro and In Vivo Anti-Cancer Activities. Molecules.

[99] Devi V.S., Rao M. (2014). *Alpinia speciosa*: A Gold Ornamental Plant—A Review. World J. Pharm. Res..

[100] Chan E.W.C., Wong S.K., Chan H.T. (2017). *Alpinia zerumbet*, a Ginger Plant with a Multitude of Medicinal Properties: An Update on Its Research Findings. J. Chin. Pharm. Sci..

[101] Kumar A., Bind V. (2018). *Alpinia zerumbet* an Essential Medicinal Herb. MOJ Toxicol..

[102] Xiao T., Huang J., Wang X., Wu L., Zhou X., Jiang F., He Z., Guo Q., Tao L., Shen X. (2020). *Alpinia zerumbet* and Its Potential Use as an Herbal Medication for Atherosclerosis: Mechanistic Insights from Cell and Rodent Studies. Lifestyle Genom..

[103] Chan E.W.C., Kezuka M., Chan H.T., Wong S.K. (2023). *Alpinia zerumbet*: A Review of the Chemistry, Quantity, and Pharmacological Properties of Selected Kavalactones. J. Nat. Rem..

[104] Kimura Y., Takido M., Nakano K., Takishita M. (1966). Studies on the Constituents of Alpinia. X: On the Constituents of the Rhizomata of *Alpinia speciosa* K. SCHUMANN and *A. kumatake* MAKINO (*A. formosana* K. SCHUMANN). Yakugaku Zasshi.

[105] Itokawa H., Morita M., Mihashi S. (1981). Phenolic Compounds from the Rhizomes of *Alpinia speciosa*. Phytochemistry.

[106] Chen J.J., Liao H.R., Chen K.S., Cheng M.J., Shu C.W., Sung P.J., Lim Y.P., Wang T.C., Kuo W.L. (2017). A New 2*H*-Pyran-2-One Derivative and Anti-Inflammatory Constituents of *Alpinia zerumbet*. Chem. Nat. Compd..

[107] Rao Y.K., Shih H.N., Lee Y.C., Cheng W.T., Hung H.C., Wang H.C., Chen C.J., Tzeng Y.M., Lee M.J. (2014). Purification of Kavalactones from *Alpinia zerumbet* and Their Protective Actions against Hydrogen Peroxide-Induced Cytotoxicity in PC12 Cells. J. Biosci. Bioeng..

[108] Nishidono Y., Iwama Y., Shirako S., Ishii T., Okuyama T., Nishizawa M., Tanaka K. (2023). Two New Monoterpene Esters from the Pericarps of *Alpinia zerumbet*. Nat. Prod. Res..

[109] Ohtsuki T., Kikuchi H., Koyano T., Kowithayakorn T., Sakai T., Ishibashi M. (2009). Death Receptor 5 Promoter-Enhancing Compounds Isolated from *Catimbium speciosum* and Their Enhancement Effect on TRAIL-Induced Apoptosis. Bioorg. Med. Chem..

[110] Fujita T., Nishimura H., Kaburagi K., Mizutani J. (1994). Plant Growth Inhibiting α-Pyrones from *Alpinia speciosa*. Phytochemistry.

[111] You H., He M., Pan D., Fang G., Chen Y., Zhang X., Shen X., Zhang N. (2022). Kavalactones Isolated from *Alpinia zerumbet* (Pers.) Burtt. et Smith with Protective Effects against Human Umbilical Vein Endothelial Cell Damage Induced by High Glucose. Nat. Prod. Res..

[112] Krishna B.M., Chaganty R.B. (1973). Cardamonin and Alpinetin from the Seeds of *Alpinia speciosa*. Phytochemistry.

[113] Natsume N., Yonezawa T., Woo J.T., Teruya T. (2021). Effect of Pinocembrin Isolated from *Alpinia zerumbet* on Osteoblast Differentiation. Cytotechnology.

[114] Taira N., Nguyen B.C.Q., Tawata S. (2017). Hair Growth Promoting and Anticancer Effects of P21-Activated Kinase 1 (PAK1) Inhibitors Isolated from Different Parts of *Alpinia zerumbet*. Molecules.

[115] Xiong T., Zeng J., Chen L., Wang L., Gao J., Huang L., Xu J., Wang Y., He X. (2023). Anti-Inflammatory Terpenoids from the Rhizomes of Shell Ginger. J. Agric. Food Chem..

[116] Be Tu P.T., Chompoo J., Tawata S. (2015). Hispidin and Related Herbal Compounds from *Alpinia zerumbet* Inhibit Both PAK1-Dependent Melanogenesis in Melanocytes and Reactive Oxygen Species (ROS) Production in Adipocytes. Drug Discov. Ther..

[117] Xu H.X., Dong H., Sim K.Y. (1996). Labdane Diterpenes from *Alpinia zerumbet*. Phytochemistry.

[118] Morita M., Nakanishi H., Morita H., Mihashi S., Itokawa H. (1996). Structures and Spasmolytic Activities of Derivatives from Sesquiterpenes of *Alpinia speciosa* and *Alpinia japonica*. Chem. Pharm. Bull..

[119] Zhang Y., Yu Y.Y., Peng F., Duan W.T., Wu C.H., Li H.T., Zhang X.F., Shi Y.S. (2021). Neolignans and Diarylheptanoids with Anti-Inflammatory Activity from the Rhizomes of *Alpinia zerumbet*. J. Agric. Food. Chem..

[120] Masuda T., Mizuguchi S., Tanaka T., Iritani K., Takeda Y., Yonemori S. (2000). Isolation and Structure Determination of New Antioxidative Ferulic Acid Glucoside Esters from the Rhizome of *Alpinia speciosa*, a Zingiberaceae Plant Used in Okinawan Food Culture. J. Agric. Food Chem..

[121] Bilia A.R., Scalise L., Bergonzi M.C., Vincieri F.F. (2004). Analysis of Kavalactones from *Piper methysticum* (Kava-Kava). J. Chromatogr. B.

[122] Xuan T.D., Teschke R. (2015). Dihydro-5,6-Dehydrokavain (DDK) from *Alpinia zerumbet*: Its Isolation, Synthesis, and Characterization. Molecules.

[123] Van H.T., Thang T.D., Luu T.N., Doan V.D. (2021). An Overview of the Chemical Composition and Biological Activities of Essential Oils from *Alpinia* Genus (Zingiberaceae). RSC Adv..

[124] Luz A.I.R., Zoghbi M.G.B., Ramos L.S., Maia J.G.S., Silva M.L. (1984). Essential Oils of Some Amazonian Zingiberaceas, 3. Genera *Alpinia* and *Rengalmia*. J. Nat. Prod..

[125] Victório C.P., da Silva Riehl C.A., Lage C.L.S. (2009). Simultaneous Distillation-Extraction, Hydrodistillation and Static Headspace Methods for the Analysis of Volatile Secondary Metabolites of *Alpinia zerumbet* (Pers.) Burtt et Smith. from Southeast Brazil. J. Essent. Oil-Bear Plants.

[126] Victório C.P., Leitão S.G., Lage C.L.S. (2010). Chemical Composition of the Leaf Oils of *Alpinia zerumbet* (Pers.) Burtt et Smith and *A. purpurata* (Vieill) K. Schum. From Rio de Janeiro, Brazil. J. Essent. Oil Res..

[127] dos Santos J.V.B., de Almeida Chaves D.S., de Souza M.A.A., Riger C.J., Lambert M.M., Campos D.R., Moreira L.O., dos Santos Siqueira R.C., de Paulo Osorio R., Boylan F. (2020). In Vitro Activity of Essential Oils against Adult and Immature Stages of *Ctenocephalides felis felis*. Parasitology.

[128] Brandão R.M., Cardoso M.G., de Oliveira J.E., Barbosa R.B., Ferreira V.R.F., Campolina G.A., Martins M.A., Nelson D.L., Batista L.R. (2022). Antifungal and Antiocratoxigenic Potential of *Alpinia speciosa* and *Cymbopogon flexuosus* Essential Oils Encapsulated in Poly(Lactic Acid) Nanofibres against *Aspergillus* Fungi. Lett. Appl. Microbiol..

[129] Kawai H., Kuraya E., Touyama A., Higa O., Hokamoto K., Tokeshi K., Yasuda A., Naragaki T., Itoh S. (2021). Improved Yield and Antioxidant Activity of Essential Oil from *Alpinia zerumbet* (Zingiberaceae) Leaves by Underwater Shockwave Pretreatment. Food Bioprod. Process..

[130] Padalia R.C., Chanotiya C.S., Sundaresana V. (2010). Compositional Variability in Essential Oil from Different Parts of *Alpinia speciosa* from India. Nat. Prod. Commun..

[131] Saikia J., Sarkar A., Washmin N., Borah T., Das B., Konwar P., Siga A., Banik D. (2023). Effect of Postharvest Drying on Physicochemical Properties, Volatile Yield, Composition, and Sensory Attributes of *Alpinia zerumbet* (Shell Ginger) Rhizome. Ind. Crops Prod..

[132] Dũng N.X., Chính T.D., Rãng D.D., Leclercq P.A. (1994). Constituents of the Flower Oil of *Alpinia speciosa* K. Schum. from Vietnam. J. Essent. Oil Res..

[133] Joshi S., Prakash O., Pant A.K., Mathela C.S. (2010). Chemical Composition, and Antioxidant and Antimicrobial Activities of *Alpinia nutans* Rosc. J. Essent. Oil Res..

[134] Luz J.G.R., Nogueira J.N., Alves C.M.G., Videira M.N., Canuto K.M., Castro K.N.C., Tavares-Dias M. (2021). Essential Oil of *Alpinia zerumbet* (Zingiberaceae) Has Anthelmintic Efficacy against Monogenean of *Colossoma Macropomum* (Characiformes: Serrasalmidae). Aquac. Res..

[135] Santos B.A., Roman-Campos D., Carvalho M.S., Miranda F.M.F., Carneiro D.C., Cavalcante P.H., Cândido E.A.F., Filho L.X., Cruz J.S., Gondim A.N.S. (2011). Cardiodepressive Effect Elicited by the Essential Oil of *Alpinia speciosa* Is Related to L-Type Ca2+ Current Blockade. Phytomedicine.

[136] Adams R.P. (2007). Identification of Essential Oil Components by Gas Chromatography/Mass Spectrometry.

[137] Padalia R.C., Verma R.S., Sundaresan V., Chanotiya C.S. (2010). Chemical Diversity in the Genus *Alpinia* (Zingiberaceae): Comparative Composition of Four *Alpinia* Species Grown in Northern India. Chem. Biodivers..

[138] Tawata S., Taira S., Kobamoto N., Ishihara M., Toyama S. (1996). Syntheses and Biological Activities of Dihydro-5,6-Dehydrokawain Derivatives. Biosci. Biotechnol. Biochem..

[139] Elzaawely A.A., Xuan T.D., Tawata S. (2007). Changes in Essential Oil, Kava Pyrones and Total Phenolics of *Alpinia zerumbet* (Pers.) B.L. Burtt. & R.M. Sm. Leaves Exposed to Copper Sulphate. Environ. Exp. Bot..

[140] Victório C.P., Lage C.L.S., Kuster R.M. (2009). Flavonoid Extraction from *Alpinia zerumbet* (Pers.) Burtt et Smith Leaves Using Different Techniques and Solvents. Eclética Química.

[141] Victório C.P., Lage C.L.S., Kuster R.M. (2010). Flavonoids Extraction from *Alpinia zerumbet* (Pers.) Burtt et Smith Leaves Using Different Procedures. Eclética Química.

[142] Kuster R.M., Mpalantinos M.A., De Holanda M.C., Lima P., Brand E.T., Parente J.P. (1999). GC-MS Determination of Kava-Pyrones in *Alpinia zerumbet* Leaves. J. High Resolut. Chromatogr..

